# A simple Fourier filter for suppression of the missing wedge ray artefacts in single-axis electron tomographic reconstructions

**DOI:** 10.1016/j.jsb.2014.02.004

**Published:** 2014-04

**Authors:** Lubomír Kováčik, Sami Kerïeche, Johanna L. Höög, Pavel Jůda, Pavel Matula, Ivan Raška

**Affiliations:** a Charles University in Prague, First Faculty of Medicine, Institute of Cellular Biology and Pathology, Albertov 4, 128 01 Prague 2, Czech Republic; b Sir William Dunn School of Pathology, University of Oxford, South Parks Road, Oxford OX1 3RE, UK; c MPI-CBG, Photenhauerstr. 108, 01307 Dresden, Germany; d Centre for Biomedical Image Analysis, Faculty of Informatics, Masaryk University, Brno, Czech Republic

**Keywords:** Electron tomography, Missing wedge, Missing wedge artefacts, Single-axis tilting

## Abstract

The limited specimen tilting range that is typically available in electron tomography gives rise to a region in the Fourier space of the reconstructed object where experimental data are unavailable – the missing wedge. Since this region is sharply delimited from the area of available data, the reconstructed signal is typically hampered by convolution with its impulse response, which gives rise to the well-known missing wedge artefacts in 3D reconstructions. Despite the recent progress in the field of reconstruction and regularization techniques, the missing wedge artefacts remain untreated in most current reconstruction workflows in structural biology. Therefore we have designed a simple Fourier angular filter that effectively suppresses the ray artefacts in the single-axis tilting projection acquisition scheme, making single-axis tomographic reconstructions easier to interpret in particular at low signal-to-noise ratio in acquired projections. The proposed filter can be easily incorporated into current electron tomographic reconstruction schemes.

## 1 Introduction

Three-dimensional electron tomographic reconstructions are produced by the acquisition of a set of tilted projections that are subsequently aligned and subjected to a reconstruction algorithm. Unfortunately, the computed reconstructions typically suffer from a number of artefacts arising from an imprecise projection alignment, from the structural instability of specimens during tomogram acquisition, and from the presence of the missing wedge region in the Fourier space of reconstructions, which remains free of experimental data due to the limited possibility of specimen tilting in transmission electron microscopes (TEMs). The utilizable range of specimen tilting is restricted not only by physical constraints imposed by the design of TEMs, specimen holders and grids, but also by the increasing effective thickness of specimen sections at tilting (Koster and Barcena, 2006; Penczek and Frank, 2006; Turner and Valdre, 1992). Therefore, reconstructions without the artefacts caused by the missing wedge can only be obtained when imaging needle-shaped (or other narrowly shaped specimens) mounted in dedicated holders that allow tilting in TEMs without restrictions (Kawase et al., 2007).

**Fig.1:**
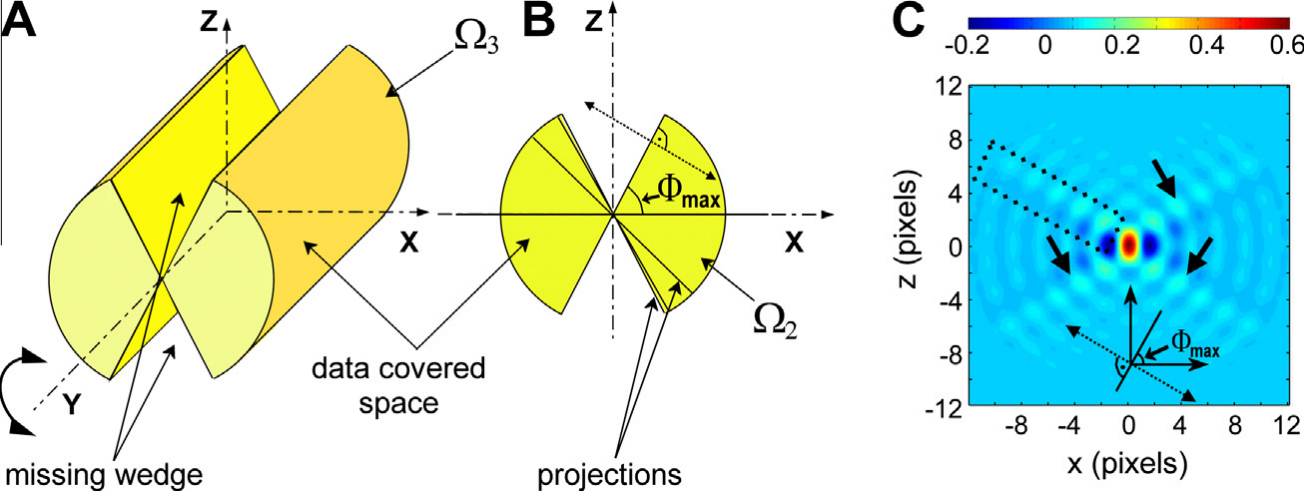
The geometric shape of areas of available data at the single axis tilting scheme with a tilting range limited to ±*Φ*_max_ in Cartesian coordinates. (A) Area *Ω*_3_(*X*,*Y*,*Z*) in 3D space, (B) *Ω*_2_(*X*,*Z*) in 2 dimensions. (C) Impulse response *W*_2_(*x*,*z*) of *Ω*_2_(*X*,*Z*) shown in (B) computed according to (Komrska, 1983). Values of *W*_2_(*x*,*z*) were divided by the magnitude in the central maximum of the impulse response of an unobstructed (full) circle with the same diameter. Dotted rectangle encloses one of the four side rays pairs perpendicular to the highest-tilt projections (light blue areas correspond to the positive line, dark blue to the negative line), arrows indicate the remaining three pairs of side rays.

At the single-axis tilting tomographic acquisition scheme, with a tilting range bounded by the highest-tilt angles *Φ*_min_ and *Φ*_max_, the missing wedge has a form of a sharply delimited double V-shaped region. Consequently, the region of available data is represented by a cylinder with the missing wedge (area marked *Ω*_3_(*R*,*Φ*,*Z*) in Fig.1A), supposing that Fourier transform values at all spatial frequencies within the angular range <*Φ*_min_, *Φ*_max_> up to some maximum spatial frequency *R*_max_ can be estimated. In two-dimensional reconstructions, *Ω*_3_(*R*,*Φ*,*Z*) reduces to a butterfly-shaped area *Ω*_2_(*R*,*Φ*) (Fig.1B) defined as

(1) $$\Omega_{2}(R\text{,}\Phi )=1\;\mathrm{if}|R|\leq R_{\text{max}}\text{,}\;\Phi_{\text{min}}\leq \Phi \leq \Phi_{\text{max}}\text{,and}0\;\text{otherwise.}$$

Its Fourier transformation leads to the well-known impulse response of the missing wedge *W*_2_(*r*,*φ*) (Fig.1C) (Carazo, 1992; Radermacher, 1988; Tam and Perez-Mendez, 1981a,b), which convolves with the reconstructed signal, giving rise to three kinds of distinctive artefacts: (i) the ray artefacts of relatively low intensity but infinite in length, which are perpendicular to each highest-tilt projection, (ii) the elongation of reconstructed features in the direction of the axis of the missing wedge (≡*z* axis in most 3D reconstructions), and (iii) the side minima joining the central spot in the direction of the *x*-axis.

The standard reconstruction methods currently used in electron tomography in structural/cell biology – the weighted back-projection (WBP), (Radermacher, 1992), the direct Fourier methods (DFM) (Crowther et al., 1970; Lanzavecchia et al., 1993; Natterer, 1985), and the iterative methods ART (Gordon et al., 1970) and SIRT (Gilbert, 1972) – can determine only a small amount of Fourier coefficients in the missing wedge in close proximity of the tilting axis, see e.g. (Lee et al., 2008). Therefore, a major portion of the missing wedge region remains empty, which gives rise to the missing wedge artefacts that make the interpretation of reconstructions difficult (e.g. Quinto et al., 2009; Radermacher, 2006), especially at low signal-to-noise ratios in projections of crowded environments of in situ specimens (Forster et al., 2005; Frangakis and Hegerl, 2006; Frangakis et al., 2002; Grünewald et al., 2003).

The missing wedge artefacts can be mitigated if regularization terms are incorporated into the reconstruction schemes (Carazo, 1992; Fanelli and Oktem, 2008; Norlen et al., 2009; Penczek and Frank, 2006; Quinto et al., 2009). While the standard reconstruction methods already regularize the solution of the severely ill-posed reconstruction problem (Fanelli and Oktem, 2008; Norlen et al., 2009), further improvement of the reconstruction quality can be achieved if it is possible to introduce some kind of *a priori* knowledge about specimens under study into the reconstruction schemes. Methods like the total variation minimization (TV) (Lu et al., 2010; Persson et al., 2001; Aganj et al., 2007), constrained maximum entropy tomography (Skoglund et al., 1996), discrete tomography (Batenburg et al., 2009), the HECT reconstruction method (Jarisch, 2010), and especially the equally-sloped tomography (EST) (Miao et al., 2005) have demonstrated their efficiency for a variety of specimen types, including cryo-tomograms of cells (Aganj et al., 2007; Lee et al., 2008). Unlike the regularization methods, the electron lambda-tomography reconstruction method (ELT) (Quinto et al., 2009) does not require any specific knowledge about specimens but reduces in particular the background clutter in reconstructions through minimization of the interference of information from structures outside the reconstructed region of interest.

Indeed, the missing wedge artefacts can be reduced or even completely avoided if the missing wedge is filled with the appropriate projection data. A full elimination of the missing wedge can be achieved by symmetry operations if the reconstructed object has a symmetry, or by sub-tomogram averaging of repetitive cellular structures or of multiple copies of randomly oriented identical particles (Bartesaghi et al., 2008; Forster and Hegerl, 2007; Frangakis et al., 2002; Ofverstedt et al., 1997). Reduction of the missing wedge area is being routinely achieved by recording of two or more tilt series of projections around different tilting axes in double-axis tilting tomography (e.g. (Mastronarde, 1997; Penczek et al., 1995)) or multiple-axis tilting tomography (Messaoudii et al., 2007). In conical tomography (Lanzavecchia et al., 2005; Zampighi et al., 2005), the missing wedge is reduced to a missing cone after specimen tilting followed by in-plane rotations. All these techniques lead to suppression of the missing wedge artefacts in reconstructions (Mastronarde, 1997), however, specimens might be exposed to higher electron doses.

In low-dose cryo-electron tomography in structural/cell biology, reconstructions are nowadays commonly computed by WBP, ART or SIRT and then subjected to various denoising procedures in order to facilitate their interpretation (Fernandez, 2012; Frangakis and Hegerl, 2006; Narasimha et al., 2008). These methods, including the efficient non-linear anisotropic diffusion (NAD) (Frangakis and Hegerl, 2001), however, do not specifically aim at suppression of the missing wedge artefacts. Therefore we propose a simple angular filter for single-axis tilting tomographic reconstructions, which efficiently suppresses the missing wedge ray artefacts by damping the sharp transition of the non-zero data region to the zero-filled missing wedge region in the Fourier space of the reconstructed object. The removal of the rays simplifies the interpretation of reconstructed volumes, in particular in sections perpendicular to the tilting axis.

## 2 Theory

In the theory of Fraunhofer diffraction, the Abbe theorem (Komrska, 1983; Straubel, 1895) states that each straight edge of a diffraction shade gives rise to an intensity line in the Fraunhofer diffraction pattern, which is perpendicular to this edge and passes through the central spot of the diffraction pattern. In the case of a tomographic reconstruction of a two-dimensional object from an angularly limited set of projections, the data and the missing wedge regions are in its Fourier space sharply separated by the lines of both highest-tilt projections (Fig.1B). The data area *Ω*_2_(*R*, *Φ*) has a geometric shape of a 2-fold rotationally symmetric section star as defined in (Komrska, 1983), except that the angular range <*Φ*_min_, *Φ*_max_*>* of the transparent sectors of *Ω*_2_(*R*, *Φ*) is generally not limited to <−*π*/4, *π*/4*>* in electron tomographic experiments. Komrska also showed that the diffraction pattern of 2-fold section stars is real and contains four pairs of arms, which are perpendicular to the straight edges of the section stars. Each pair is composed of two adjacent intensity lines passing over the primary diffraction spot at a small distance. The analytic computation of the exact forms of Fourier transforms of section stars also provided in (Komrska, 1983) showed that (i) the four pairs of arms are the lines of the slowest intensity decrease in the diffraction pattern, (ii) one of the two lines in an arm pair had in fact positive values of Fourier transform, while the other one was negative (Fig.1C), and (iii) the central diffraction spot of the computed impulse response is elongated in the direction of the axis of the non-transparent regions of the diffraction shade (i.e. corresponding to the direction of the axis of the missing wedge). Similar result was also obtained by Tam and Perez-Mendez (1981a,b).

**Fig.2:**
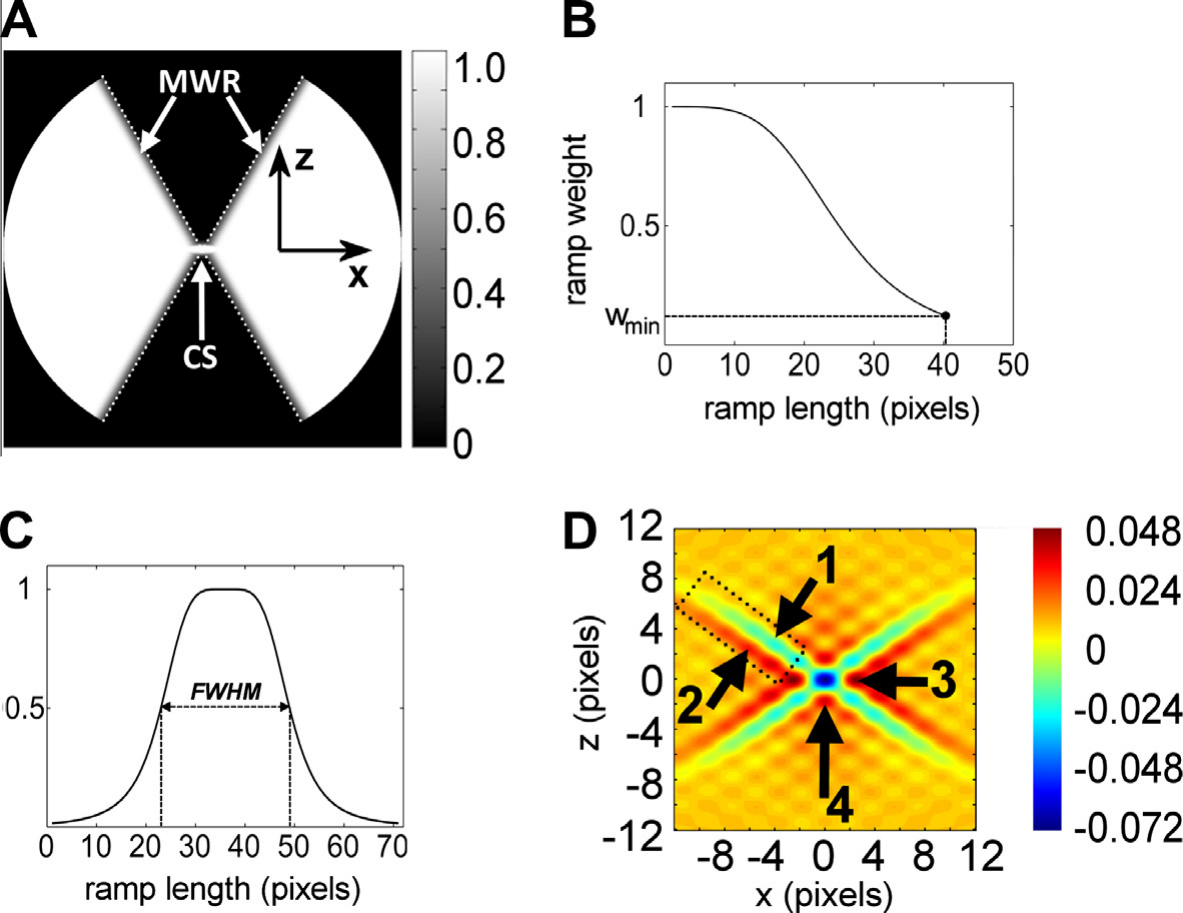
Design of an angular filter. (A) An angular filter *Ω_A_*(*X*,*Z*) derived from *Ω*_2_(*X*,Z) shown in Fig.1B. MWR-smooth missing wedge ramps inserted into the data-available area *Ω*_2_(*X*,*Z*), CS-central stripe. The dotted white lines indicate positions of both highest-tilt projections bordering the area of available data. (B) An example of a Butterworth intensity profile of the missing wedge ramp. (C) An example of a Butterworth intensity profile of the central stripe, with full-width at half-maximum (FWHM) marked by the dashed line. (D) Intensity map of the difference *W_A_*(*x*,*z*) – *W*_2_(*x*,*z*). The impulse response *W_A_*(*x*,*z*) was numerically computed from the angular filter *bfly20-4-0.2-15-4-10* and normalized in the same way as the impulse response *W*_2_(*x*,*z*) presented in Fig.1C. Note the decrease of intensities in the positive line of a side ray pair (arrow 1) and increase in the negative line (arrow 2), and higher intensities in the side minima along both x- and z-axes (arrows 3 and 4). The dotted black rectangle indicates the position of the same side-rays pair as in Fig.1C.

In the single-axis-tilting electron tomography, the impulse response *W*_3_(*r*,*φ*,*z*) of the three-dimensional data-available area *Ω*_3_(*R*,*Φ*,*Z*) reduces to a two-dimensional pattern *W*_2_(*r*,*φ*) because the data region *Ω*_3_(*R*,*Φ*,*Z*) uniformly spans the whole period of the discretely sampled Fourier space along the tilting axis. The intensity rays produced by the side arms of *W*_2_(*r*,*φ*) span a substantial portion of the reconstructed volumes, where they interfere with reconstructed structures or may create false structural features. However, if the transition between the data and the missing wedge regions is forced to be smoother, the intensity of the side arms of the impulse response decreases (Fig.2D).

## 3 Material and methods

### 3.1 Design of angular filters in Cartesian coordinates

The angular filter *Ω_A_*(*X*,*Z*) has to meet two goals:

1. attenuate the intensity step between the data and the missing wedge regions, and
2. keep Fourier coefficients at the lowest spatial frequencies unchanged to prevent high-pass filtering effects.

The intensity step between the data and the missing wedge regions can be simply attenuated by replacement of the unitary intensities in small regions of the data-available area *Ω*_2_(*X*,*Z*) adjacent to the lines of both highest-tilt projections by four smooth weighting ramps (Fig.2A) whose gradient is oriented perpendicularly to the lines of the highest-tilt projections. The profile of the weighting ramps can take a form of any standard low-pass filter, e.g. the Gaussian or the Butterworth filter (Fig.2B). The weights of the profile are assigned values in interval <*w*_min_,1>, where the lowest weight *w*_min_ is applied to the highest-tilt projections and the weight of 1 is placed into the data-available area. A missing-wedge filter *Ω_MW_*(*X*,*Z*) designed this way would attenuate intensities of all rays in the direction of the gradient of the weighting ramps, however, it would also down-weight low spatial frequencies in the region close to the tilting axis, which would lead to undesirable high-pass filtering effects. Therefore, a protective two-sided weighting ramp *CS*(*X*,*Z*) (*CS*: central stripe) has to be placed along the *X* axis of *Ω_MW_*, which has a value of 1 at *Z* = 0 and decreases with |*Z|*. This ramp can also take the form of the standard low-pass filters (Fig.2C). The angular Fourier filter *Ω_A_*(*X*,*Z*) shown in Fig.2A is then computed as max(*Ω_MW_*(*X*,*Z*), *CS*(*X*,*Z*))*.*

The strength of the filter can be modified by changing the width of the ramps and the weight at the highest-tilt projection *w*_min_. The ray artefacts are usually substantially reduced when *w*_min_ is set to ∼0.2, the length of the ramp is set to ∼16–64 pixels, and when the ramp has a Butterworth profile of the 2nd or 4th order. The form and the length of the *CS* ramp have to be optimized for each reconstruction so that the *CS* ramp sufficiently prevents the missing wedge ramps from attenuating the signal at the lowest spatial frequencies in the vicinity of the tilting axis. When the designed central stripe is too narrow, high-pass filtering effects and strong resolution loss in the direction of the *z*-axis occur. Conversely, if the central stripe is too broad, the ray suppression may be weak. In most applications, suitable central stripe ramps are formed by two opposing Butterworth profiles of the 2nd or 4th orders yielding a ramp with full-widths at half-maximum (FWHM) ranging from 20 to 50 pixels.

A similar filter, however without the central stripe for the protection of the lowest spatial frequencies, had been invented for an improved data storage at spiral computerized tomography (Gruebnau and Stierstorfer, 2004).

### 3.2 Filter naming convention

Due to the butterfly shape of the angular filter, the angular filters will be in the following text named “bfly” followed by two sets of hyphen-separated parameters describing the profiles of the missing wedge ramp and of the central stripe ramp. All filters used in this study had Butterworth profiles, therefore the missing wedge ramp is described by the length of the ramp, the order of the Butterworth profile, and the weight at the highest-tilt projection. The description for the central stripe is the same except that the weight at the highest-tilt projection is replaced by the cutoff (half-width at half-maximum) of the Butterworth profile so that control of the width of the stripe is provided. Parameters of ramp profiles for angular filters used in this study are listed in Table 1.

### 3.3 Evaluation of the reduction of intensity variances in outer regions of impulse responses of angular filters

In order to estimate the ability of angular filters to reduce the background clutter, impulse responses *W_A_*(*x*,*z*) of all angular filters used in this study and of the impulse response *W*_2_(*x*,*z*) were numerically computed and variances *σ*^2^ of their intensities in an annular region *AR*(*x*,*z*) around their central peaks were estimated. Inner radius of the annulus *AR* was 2 pixels, outer radius was 25 pixels, and the annulus was centered at *x* = 0, *z* = 0. In this way, the central peak was excluded from the variance estimation. Ratios of intensity variances of the impulse responses *W_A_*(*x*,*z*) and *W*_2_(*x*,*z*) within the annulus *AR*.

$$r_{A}=\frac{\sigma^{2}(W_{A}(\mathrm{AR}(x\text{,}z)))}{\sigma^{2}(W_{2}(\mathrm{AR}(x\text{,}z)))}$$

reflect the background smoothing ability of angular filters. The values *r_A_* for all angular filters used in this study are listed in Table 1.

### 3.4 Test volumes for quantitative analysis of angular filtering

**Fig.3:**
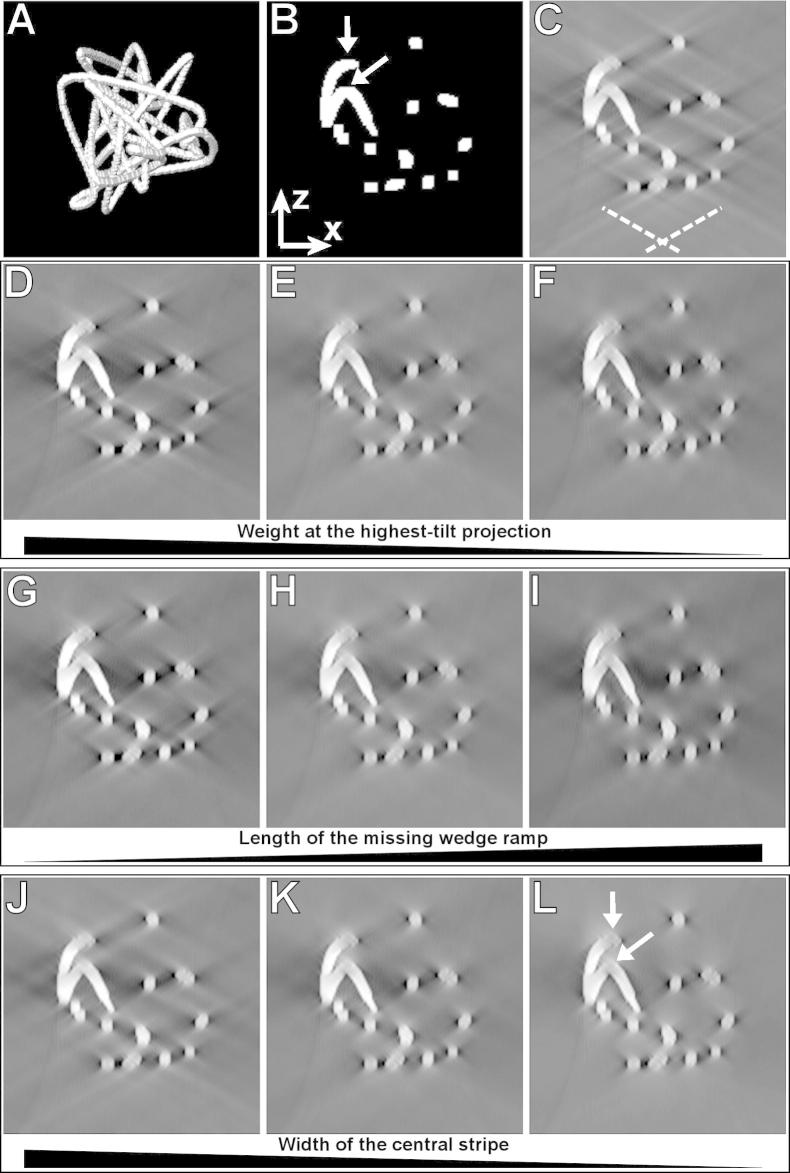
Results of angular filtering of a volume containing a random knot with unitary intensity. (A) A view at the test random knot. (B) An *x*–*z* cross-section (perpendicular to the tilting axis) through the test volume. (C) The cross-section shown in (B) after reconstruction from 121 noise-free projections computed within the ±60° range at a 1° step. Dashed lines indicate the directions of the side rays. (D) – (L): Results of angular filtering with different filters demonstrated on the cross-section shown in (B). (D) – (F): Filtering with decreasing weight of the missing wedge ramp at the highest-tilt projection. Angular filters used were *bfly20-4-**0.5**-15-4-10* in (D), *bfly20-4-**0.2-**15-4-10* in (E), and *bfly20-4-**0.13-**15-4-10* in (F). (G) – (I): Filtering with increasing length of the missing wedge ramp. Angular filters used were *bfly**10-**4-0.2-15-4-10* in (G), *bfly**20-**4-0.2-15-4-10* in (H), and *bfly**40-**4-0.2-15-4-10* in (I). (J) – (L): Filtering with decreasing width of the central stripe. Angular filters used were *bfly20-4-0.2-**25-4-20*** in (J), *bfly20-4-0.2-**15-4-10*** in (K), and *bfly20-4-0.2-**8-2-4*** in (L). Arrows show locations with a decreased intensity of horizontal lines after angular filtering with a narrow central stripe.

We used three kinds of phantom volumes for the quantitative evaluation of the effect of angular filtering, all 151 × 151 × 151 voxels large. The first type was the random cylinders volume, which contained 40 randomly placed cylinders of unitary intensity. Each cylinder was assigned a random height and a diameter in the range of 3–20 pixels and its rotational axis was set parallel to one of the *x*, *y*, and *z* axes. Secondly, random 3D knots with 40 nodes made of lines 6 pixels thick were generated according to (Bellon et al., 1998). The signal in the lines was constant, of unitary magnitude. In the third modification, cross-sections of the 6-pixel knot lines were modified to have a 2D Gaussian intensity profile with *σ* = 1.8 pixels, and additionally 10 small cylinders of intensities equal to 3× the mean knot line intensity were randomly placed into the volumes in the same way as in the random cylinders volumes in order to simulate features of extreme intensities such as colloidal gold balls. The diameters of the added cylinders ranged from 3 to 10 pixels, their height from 2 to 5 pixels. An example of the random knot test volume is illustrated in Fig.3A. All test volumes were created in Matlab (Mathworks, Natick, MA, USA).

Projections of all test volumes were computed with a 1° step in the ±60° range and either no noise or Gaussian noise was added to projections with signal-to-noise ratios (SNR) of 0.01, 0.1, 0.5, 1, and 5. 3D reconstructions were computed by bilinear interpolation in the Fourier space. In addition, the random cylinders volumes were also reconstructed by 30 SIRT iterations as implemented in TomoJ (Messaoudii et al., 2007). After reconstruction, the test volumes were treated by all angular filters listed in Table 1.

### 3.5 Evaluation of quality of reconstructed volumes

Quality of the reconstructed volumes was evaluated by Figures of Merit (FOMs) (Marabini et al., 1997; Sorzano et al., 2001). Structural consistency was evaluated by error FOMs (eFOMs) (Marabini et al., 1997), density standard deviation FOMs (scσFOMs) and range FOMs defined as in (Sorzano et al., 2001), computed against the model volumes. Structural separability was estimated by the foreground mean separability FOMs (hsFOMs) and detectability error FOMs (hsdtFOMs) as defined in (Sorzano et al., 2001). eFOMs and scσFOMs were computed both for the full volumes 151 × 151 × 151 voxels large (whole volume eFOMs) as well as separately for signal voxels (signal voxels eFOMs, signal voxels scσFOMs) and background voxels (background voxels eFOMs, background voxels scσFOMs). The foreground mean separability FOMs were calculated for intensities in all signal voxels against all background voxels. SNRs in reconstructions were computed as *μ*_sv_/*σ*_bv_, where *μ*_sv_ is the mean intensity of signal voxels and *σ*_bv_ is the standard deviation of background voxels. The non-zero voxels in the noise-free phantoms were considered to be the signal voxels, the zero voxels were then the background voxels. 10 phantom volumes of each kind were generated at each SNR in projections. All FOMs were computed in Matlab (Mathworks, Natick, MA, USA).

### 3.6 Evaluation of performance of angular filtering

The performance of angular filtering was evaluated by *t*-tests of FOMs of angularly filtered reconstructions against the FOMs of the corresponding angularly unfiltered reconstructions, and by the mean relevancies of improvement (MRI) of the computed FOMs (Marabini et al., 1997). The MRI of an FOM is defined as:

$$\text{MRI}=100\times \frac{f_{2}-f_{1}}{1-f_{1}}$$

where *f*_1_ is the average value of the given FOM of the angularly unfiltered reconstructions and *f*_2_ is the average FOM value of their angularly filtered counterparts. The value of MRIs ranges from 0 to 100, and MRIs greater than 5 are often considered to be relevant (Marabini et al., 1997). Improvement of SNR in reconstructions and improvement of the foreground mean separability FOMs was computed as the ratio of mean SNRs and hsFOMs, respectively.

### 3.7 Application of the angular filter to tomographic datasets

The proposed angular filter can be applied either consecutively to all *X*–*Z* planes of Fourier transformed reconstructions by simple multiplication or to aligned stacks of projections prior to 3D reconstruction. In this case, each row (*Y* line) of a Fourier-transformed projection, which is perpendicular to the tilting axis, is multiplied by an intensity profile of a central section through the angular filter Ω*_A_*(*X*, *Z*) at the tilting angle of the projection. Since the geometry of the missing wedge in the Fourier space of the reconstructed object is independent of the *Y* position at the single-axis tilting acquisition scheme, angular filtering can be parallelized to speed up its application.

### 3.8 Room temperature electron tomography of hazel pollen grains

Reconstructions of structures in the wall of hazel pollen grains were obtained in the electron tomographic experiments described in (Kovacik et al., 2009). Briefly, hazel pollen grains were chemically fixed, stained by Alcian blue, epon-embedded and postfixed with osmium tetroxide and lanthanum nitrate. Electron tomography of ∼150 nm sections coated with a ∼3 nm thick fine-grained Pt/C layer deposited perpendicular to the sections by electron-beam evaporation was carried out at low temperature (∼−170 °C) with a Tecnai G2 Polara transmission electron microscope (FEI, Hillsboro, OR, USA) equipped with a Gatan energy filter (GIF 2002, Gatan, Pleasanton, USA) and a 2048 × 2048 Gatan CCD camera, operated at 300 kV. High-dose single-axis tilted series were acquired with 1° angular step in the tilting range |±65|° with ∼−6 μm nominal defocus at the magnification of 22,900×. Three-dimensional reconstructions were computed by bilinear interpolation in Fourier space and low-pass filtered with cutoff of 5 nm.

### 3.9 Cryo-electron tomography of whole cells of *Trypanosoma brucei* (*T. brucei*)

Cryo-ET tomograms were acquired and processed as in (Hoog et al., 2012). In short, low-dose single-axis tilting series of portions of whole-plunged cells of *T. brucei* were acquired at a Tecnai F30 TEM (FEI, Hillsboro, OR, USA) equipped with a 300 kV FEG, 4 K Gatan UltraCam CCD and the Tridiem Gatan Imaging Filter. 81 projections with angular spacing of 1.5° were acquired at the 27,500× magnification within the ±60° angular range in the zero-loss mode of the energy filter with an energy window of 20 eV at the nominal defocus of −6 μm. The total dose was 90e^−^/Å^2^. Reconstructions were computed in IMOD (Kremer et al., 1996) with WBP and SIRT with 30 iterations, and low-pass filtered with cutoff of 3 nm. NAD filtering was performed also in IMOD, with *k* = 0.01 and 20 iterations.

### 3.10 Room temperature electron tomography of samples prepared by pre-embedding immunogold labeling technique

Inclusions created by inhibited IMPDH2 protein known as Rings & Rods structures (Smigova et al., 2011), immunogold labeled by the pre-embedding technique, were subjected to an electron tomographic experiment. Samples were processed according to (Smigova et al., 2011). Briefly, human Hep2 cells were treated by IMPDH inhibitor ribavirin. The cells were fixed in formaldehyde, permeabilized and immunolabeled with the primary anti-IMPDH2 antibody (12948-1-AP, Proteintech, Manchester, United Kingdom) and the secondary antibody conjugated with ultrasmall gold (Aurion, Wageningen, The Netherlands). After the pre-embedding, the ultrasmall colloidal gold particles were silver enhanced, the immunolabeled cells were postfixed with glutaraldehyde, incubated with R-GENT SE-EM (Aurion), dehydrated in ethanol, embedded into Araldite/Embed 812 and polymerized. Single-axis tomographic dataset was acquired at a Tecnai T20 TEM (FEI, Hillsboro, OR, USA) equipped with a 200 kV LaB6 gun and a 2 K Gatan Ultrascan 1000 CCD at the ICBP, Prague. Projections were recorded within the ±65° range at 1° step at the magnification of 7800×, reconstructions were computed by WBP in IMOD (Kremer et al., 1996). Small sub-volumes containing a few silver-enhanced colloidal gold particles from this reconstruction were examined in order to test the effect of angular filtering in their neighborhood.

### 3.11 Electron tomography of the cerebellum molecular layer of adult rats

The reconstructed single-axis electron tomographic dataset was created by (Capani et al., 2001) and downloaded from the Cell Centered Database (http://ccdb.ucsd.edu/index.shtm, microscopy product ID: 22), (Martone et al., 2002). In this tomographic experiment, images were typically obtained over a range of ±60° at a magnification of 30,000×, with the final pixel size of 2 nm. Image processing and reconstruction was performed with the SUPRIM software suite (Schroeter and Bretaudiere, 1996). Small sub-volumes containing a colloidal gold particles from this reconstruction were examined in order to test the effect of angular filtering in their neighborhood.

## 4 Results

### 4.1 Expected effects of angular filtering

The expected effect of angular filtering is illustrated in Fig.2D, which shows the difference of impulse responses *W_A_*(*x*,*z*) – *W*_2_(*x*,*z*), where *W_A_*(*x*,*z*) is the impulse response of the angular filter *Ω_A_*(*X*,*Z*): *bfly20-4-0.2-15-4-10*. Fig.2D indicates that

(i) intensities in the positive lines of the side ray pairs are smaller along the whole length of the rays (arrow 1), whereas intensities in the negative lines of the side ray pairs are higher (arrow 2), which implies the suppression of the side rays,
(ii) there are higher intensities along both *x*- and *z*-axes in the vicinity of the central peak of the impulse response *W_A_*(*x*,*z*) (arrows 3 and 4), which suggests further prolongation of reconstructed structures along the *z*-axis and suppression of side minima along the *x*-axis, and
(iii) the magnitude of the central peak of *W_A_*(*x*,*z*) is reduced in comparison to *W*_2_(*x*,*z*).

Furthermore, Table 1 indicates that

(i) intensity fluctuations in the annular region outside of the central peak of the impulse responses *W_A_*(*x*,*z*) are smaller than intensity fluctuations in the same region of the missing wedge impulse response *W*_2_(*x*,*z*), and
(ii) that filters with lower weights at the highest-tilt projections and longer missing wedge ramps have smoother impulse responses.

Therefore, angular filtering will suppress side rays and reduce the overall background clutter in single-axis electron tomographic reconstructions, at the cost of further loss of resolution of reconstructed structures in the *z*-direction.

### 4.2 Angular filtering of phantom volumes

Fig.3 shows the effect of angular filtering with filters listed in Table 1 on a reconstruction of a random knot with a unitary intensity from noise-free projections. Fig.3A offers a view of a model random-knot volume, Fig.3B shows an *x*–*z* cross-section through the model (perpendicular to the tilting axis). Fig.3C shows the cross-section 3B after reconstruction from the 121 projections in the ±60° range. Fig.3D–F illustrate the effect of filtering with a decreasing weight of the missing wedge ramp at the highest tilt projection, Fig.3G–I the effect of filtering with a gradually prolonged missing wedge ramp, and Fig.3J–L the effect of filtering with a gradually narrower central stripe. All these three kinds of modifications of the angular filter design lead to a more effective suppression of side rays, which is however traded for a loss of *z*-axis resolution, especially if the central stripe is narrow (Fig.3L).

Results of the quantitative analysis provide a more accurate insight into the effects of angular filtering. Table 2 lists the mean relevancies of improvement of the computed FOMs for the DFM-reconstructed random-cylinders test volumes and supplementary Tables 1 and 2 for each kind of the DFM-reconstructed random knot volumes. Supplementary Table 3 contains the MRIs of SIRT-reconstructed random-cylinders volumes. The acquired MRIs of the DFM reconstructions indicate that:

1. Angular filtering improved the reconstruction quality in background voxels independently of the kind of the test phantom, the amount of noise added to projections or the strength of the angular filter, as indicated by increase in eFOMs and scσFOMs for background voxels. This result represents the reduction of the background clutter due to suppression of the side rays of the impulse response *W*_2_(*x*,*z*).
2. In contrast, angular filtering delivers more accurately reconstructed intensities in signal voxels only at low SNRs in projections (⩽1) for all test specimens, as indicated by the signal voxels eFOMs.
3. eFOMs computed from the full volumes (whole volume eFOM) show benefits of angular filtering for projection SNRs up to 1 in case of the random knots with Gaussian cross-sections and up to 5 for the random knots with constant intensities and for the random cylinders volumes.
4. Signal voxel standard deviation FOMs show superiority of angularly filtered reconstructions only if test objects had constant intensity. In the random knot volumes with 2D Gaussian intensity lines, angular filtering brings significantly more accurate reproductions of signal variances only at low SNRs in projections used (⩽0.5).
5. Improvement or deterioration of the signal-to-noise ratios in reconstructions depend both on the kind of the test specimen and the design of angular filer used. For the random cylinder volumes and random knots with Gaussian cross-sections, angular filtering leads to significantly higher SNRs in a large majority of angular filter designs and projection SNRs. The systematic exception was the angular filter with the tight central stripe (i.e. filter *bfly20-4-0.2-8-2-4* having half-width at half-maximum only 4 pixels). For the volumes with random knots of constant intensities, SNRs in angularly filtered reconstructions were significantly higher only with filters that either retained most of the signal in the vicinity of the tilting axis (i.e. filter *bfly20-4-0.2-25-4-20* having a broad central stripe 25 pixels long with a half-width at half-maximum of 20 pixels), or with filters which strongly suppressed the side rays by a long missing wedge ramp (the *bfly40-4-0.2-15-4-10* filter having a 40 pixels long missing wedge ramp). An increase of SNR in this type of test specimen was also observed at the lowest SNR (0.01) in projections.
6. The MRIs for the range FOMs showed results similar to the MRIs of signal voxels standard deviation FOMs described in point 4.
7. Both structural separability FOMs were, in general, significantly worse for angularly filtered test objects having constant intensity, with the exceptions of (i) the angular filter with the broad central stripe *bfly20-4-0.2-25-4-20* and (ii) the angular filter with long missing wedge ramp *bfly40-4-0.2-15-4-10* applied to reconstructions from low SNR projections. On the contrary, the random knot volumes with the Gaussian intensity profiles and added cylinders showed much better performance in structural separability, in particular in the foreground mean separability FOM where all angular filters except of the *bfly20-4-0.2-8-2-4* with the narrow central stripe and the *bfly10-4-0.2-15-4-10* with the short missing wedge ramp achieved significant improvement at most projection SNRs. At low SNRs in projections (⩽0.5) however, many of the differences in the structural separability FOMs are statistically insignificant.
8. MRIs of the detectability error FOMs, which express the error that would have been committed if the foreground pixels were segmented from the background by thresholding at a discrete intensity level (Sorzano et al., 2001), were significantly better after angular filtering only in few cases, independently of the kind of the test phantom. Most of these improvements were achieved with the angular filter with broad central stripe *bfly20-4-0.2-25-4-20*.

MRIs of the SIRT-reconstructed random-cylinder volumes (Supp. Table 3) are, in general, comparable to the MRIs of DFM-reconstructions. In contrast to DFM reconstructions however, the effectiveness of angular filtering is shifted to lower SNRs in projections. Particularly, angular filtering did not positively influence the quality of noise-free SIRT reconstructions in most of the measurements and there are only few positive MRIs at SNR = 5 in projections. In addition, MRIs of range FOMs are positive only at SNRs ⩽ 0.1 in projections. On the other hand however, angular filtering promoted MRIs of both structural separability FOMs of the SIRT-reconstructed volumes. Interestingly, the filter with the narrow central stripe (*bfly20-4-0.2-8-2-4*) performed best in many cases.

In summary, the quantitative results correspond well to the expected and visual behavior of the angular filter presented in Fig.3. Comparison of MRIs of the various designs of angular filters manifest that, at a constant SNR in projections, the effect of angular filtering gets more pronounced (i) with decreasing weight of the missing wedge ramp at the highest-tilt projection and (ii) with increasing length of the missing wedge ramp. This result corresponds to the ratios of smoothing of outer region of the impulse responses of angular filters *W_A_*(*x*,*z*) presented in Table 1. Stronger side rays filtering reduces the background clutter more efficiently (MRIs of variance in background voxels increase with the strength of the filter), but leads to a worse accuracy of reconstructed signal voxel intensities at SNRs in projections higher than 0.5 (MRIs of signal voxel eFOMs decrease with the strength of the filter). However, most of the MRIs indicate that angular filtering is beneficial at SNRs in projections <1, which suggests its suitability particularly for cryo-EM tomograms. Furthermore, the computed MRIs indicate that filters having a broad central stripe are most suitable for a general application to single-axis tomograms, but that for SIRT reconstructions even filters with narrower central stripes can be beneficial.

### 4.3 Angular filtering of real specimens

**Fig.4:**
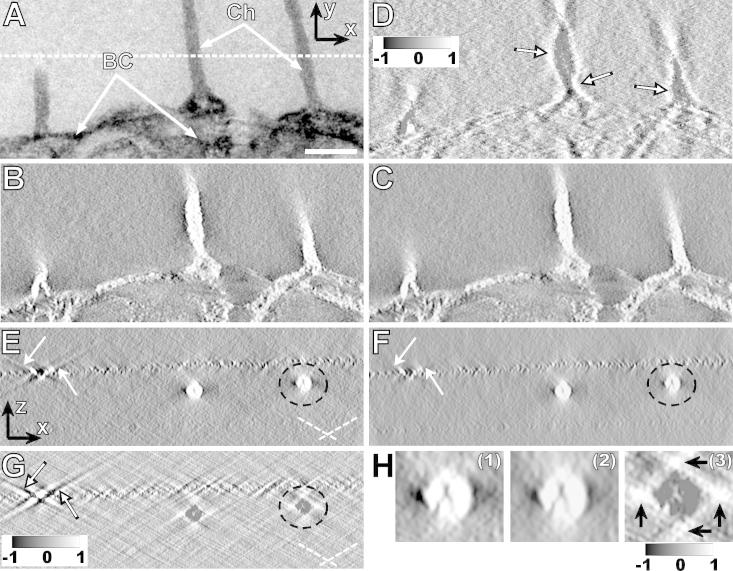
Angular filtering of hazel pollen wall reconstruction in plastic sections. (A) An untilted TEM projection showing channels (Ch) and bacula cavities (BC) in the wall of hazel pollen grains. Scale bar: 100 nm. The dashed line indicates the position of the x-z cross-section shown in (E–G). (B) An *x–y* cross-section through the reconstruction treated with a mild low-pass filter prior to angular filtering, and (C) after angular filtering. (D) Intensity difference of images (C)–(B) normalized to interval <−1, 1> showing in particular the intensity increase in the side minima (arrows). (E) An *x–z* cross-section through the reconstruction (marked in A) prior to angular filtering. Dashed lines indicate directions of the side rays. (F) The same cross-section after angular filtering. Arrows show the most intensive side-rays that are removed with angular filtering*.* (G) The intensity difference of images (F)–(E) normalized to interval <−1, 1> shows in particular the suppression of the side rays (arrows). (H) An enlarged detail marked in panels (E) – (G) by black dashed circles: (1) prior to angular filtering, (2) after angular filtering, (3) intensity difference (2)–(1). The angular filter used was *bfly48-4-0.15-24-4-12*, contrast of reconstructions was reversed for convenience prior to any other treatment. Thickness of all sections is 1.31 nm.

To test how angular filtering performed on a reconstruction of a plastic section, we used a tomogram of a hazel pollen grain containing contrasted channels and bacula cavities (Fig.4A). A tomographic *x*–*y* slice from an angularly unfiltered reconstruction shown in Fig.4B with reversed contrast appears less smooth than its angularly filtered counterpart (Fig.4C), and the difference image (Fig.4D) shows improvements in the side minima (arrows). The ray suppression is well observed primarily in the *x*–*z* cross-sections through the specimen (Fig.4E and F), in particular in the image of the intensity difference of the unfiltered and filtered cross-section (Fig.4G and H), which clearly show the expected effects of angular filtering: suppression of the side rays (arrows in Fig.4E and F), increase of intensity in side minima and prolongation of the reconstructed channels in the *z*-direction (arrows in Fig.4H^(3)^), and decrease in intensity in the reconstructed channels (Fig.4G and H). Movies 1–3 illustrate the expected effects of filtering with (1) decreasing weight at the highest-tilt projections, (2) prolonged missing wedge ramp and (3) decreasing width of the central stripe on several *x*–*z* cross-sections through the specimen.

**Fig.5:**
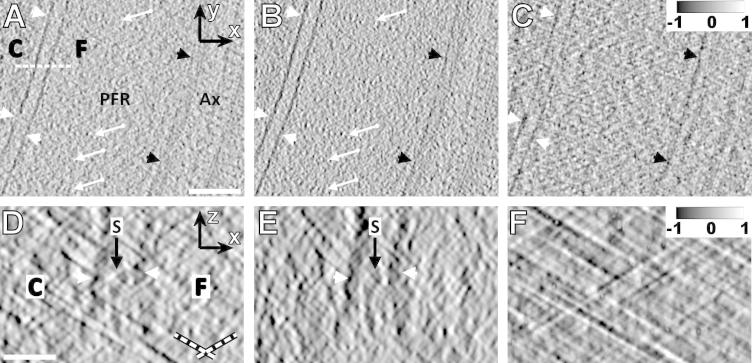
Angular filtering of the WBP-reconstructed cryo-tomogram of *T. brucei*, low-pass filtered with cutoff of 3 nm. (A) An *x–y* cross-section through NAD-filtered reconstruction. The dashed white line indicates the position of cross-sections introduced in (D). (B) The cross-section shown in (A) after angular filtering followed by NAD filtering. C-cell, F-flagellum, PFR-paraflagellar rod, Ax-axoneme. Arrows point at the periodic structures of the paraflagellar rod, white arrowheads indicate cellular membranes, and black arrowheads indicate microtubule. Scale bar: 100 nm. (C) Intensity difference of images (B)–(A) normalized to interval <−1, 1> shows in particular the suppression of grainy noise.(D) An *x–z* cross-section through NAD-filtered reconstruction. (E) The cross-section shown in (D) after angular filtering followed by NAD filtering. Arrowheads denote membranes, S stands for a staple (Hoog et al., 2012). Scale bar: 30 nm. Dashed lines in (D) indicate directions of the side rays. (F) Intensity difference of images (E)–(D) normalized to interval <−1, 1> shows in particular the suppression of the side rays. The angular filter used was *bfly65-4-0.2-30-4-10*, thickness of all sections is 5 nm.

**Fig.6:**
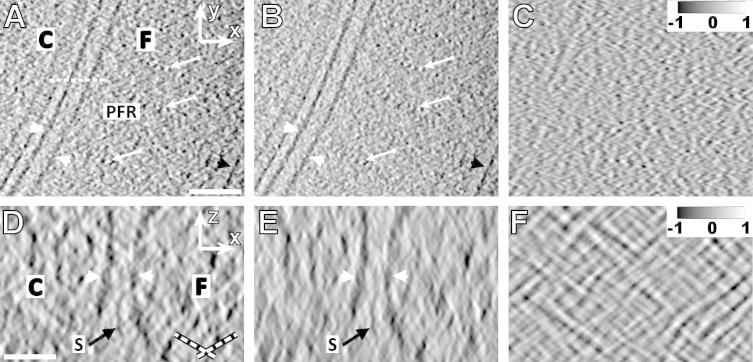
Angular filtering of the SIRT-reconstructed cryo-tomogram of *T. brucei*, low-pass filtered with cutoff of 3 nm. (A) An *x–y* cross-section through NAD-filtered reconstruction. The dashed white line indicates the position of cross-sections introduced in (D). (B) The cross-section shown in (A) after angular filtering followed by NAD filtering. C-cell, F-flagellum, PFR-paraflagellar rod. Arrows point at the periodic structures of the paraflagellar rod, white arrowheads indicate cellular membranes, and black arrowheads indicate microtubule. Scale bar: 100 nm. (C) Intensity difference of images (B)–(A) normalized to interval <−1, 1> shows in particular the suppression of grainy noise. (D) An *x–z* cross-section through NAD-filtered reconstruction. (E) The cross-section shown in (D) after angular filtering followed by NAD filtering. Arrowheads denote membranes, S stands for a staple (Hoog et al., 2012). Scale bar: 30 nm. Dashed lines in (D) indicate directions of the side rays. (F) Intensity difference of images (E)–(D) normalized to interval <−1, 1> shows in particular the suppression of the side rays. The angular filter used was *bfly65-4-0.2-30-4-10*, Thickness of all sections is 0.76 nm.

The performance of angular filtering was also tested in combination with mild low-pass and NAD filtering on a WBP and SIRT single axis cryo-electron tomographic reconstruction of *T. brucei*, where angular filtering was performed prior to NAD filtering. In the x-y planes of the angularly unfiltered WBP reconstruction, cellular and flagellar membranes appeared interrupted in places, and the repeats of the paraflagellar rod in the distal region of the axoneme and the microtubules of the axoneme were difficult to detect (Fig.5A, arrows). After angular filtering, the electron density of membrane appears more consistent, and the repeats of the PFR and the microtubules are more clearly discernable (Fig.5B). The difference image (Fig.5C) shows that in particular the grainy noise was removed from the *x*–*y* plane thanks to the angular filter. The image improvement in the WBP reconstruction is more pronounced in the *x*–*z* cross-section where the missing wedge effect is the worst. We imaged a “staple”, an electron-dense membrane spanning structure found between the cell and the flagellum (Fig.5D; Hoog et al., 2012). The application of angular filtering dramatically improved the visualization of the staple from this direction (Fig.5E). The difference image (Fig.5F) shows that the side rays produced grainy noise overlying the reconstructed structures and noticeably interfering with them. Apparently, the structures of interest in this reconstruction – membranes, staples, microtubules, and the periodic paraflagellar rod – became more pronounced after angular filtering, which is demonstrated also in Movies 4 and 5. We conclude that the angularly filtered WBP reconstruction appears clearer and sharper than its unfiltered counterpart. In the SIRT reconstructions (Fig.6), the characteristics of the tomographic noise removed by angular filtering are similar as in the WBP reconstructions (Fig.6C and F), but filtering with the same angular filter appears to have milder effects (Fig.6A, B and D, E) than in the WBP reconstruction.

### 4.4 Effect of angular filtering of sub-volumes with colloidal gold particles

**Fig.7:**
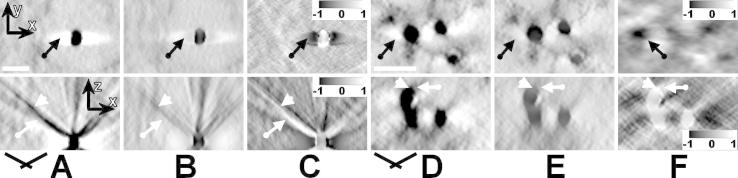
Angular filtering of colloidal gold markers. Top row: *x–y* cross-sections through reconstructed gold particles, bottom row: *x–z* cross-sections through the gold particles. (A) Cross-sections of a 20 nm colloidal gold ball obtained from the cerebellum molecular layer prior to angular filtering, (B) after angular filtering. (C) Intensity difference (B)–(A) normalized to <−1, 1>. Black arrows in the top row indicate reduction of intensity in the side maxima, white arrows in the bottom image reduction of the ray artefacts. The angular filter used was *bfly65-4-0.2-24-4-10*. (D) Cross-sections of a 20 nm silver-enhanced colloidal gold ball through the reconstruction of the immunolabeled Rings & Rods structures prior to angular filtering, (E) after angular filtering. (F) Intensity difference (E)–(D) normalized to <−1, 1>. Black arrows in the top row indicates reduction of the side maxima, white arrows in the bottom image reduction of the ray artefacts. White arrowheads indicate positions where the boundary of the gold particle was smeared due to filtering with an angular filter with a tight central stripe. The angular filter used was *bfly24-4-0.2-8-4-4*. Scale bars: 50 nm.

The diameter of the colloidal gold markers in all extracted sub-volumes ranged from 15 to 20 nm. In the *x*–*y* sections through the reconstructed gold marker from the cerebellum molecular layer (Fig.7A–C, top row), the most obvious difference is the reduction of intensity in the vicinity of the gold marker (black arrows). In the *x*–*z* sections through this marker (Fig.7A–C, bottom row), not only the most spurious streaks perpendicular to the highest-tilt projections (i.e. in the direction of angular filtering) were removed from the reconstruction (white arrows), but also intensity in streaks with small azimuthal difference was reduced (white arrowheads). Indeed, streaks with large azimuthal difference to the direction of angular filtering were not removed.

In the *x–y* sections through the silver-enhanced gold markers of the Rings & Rods structures (Fig.7D–F, top row), the effect of angular filtering is the same, i.e. reduction of intensity in the vicinity of the gold marker (black arrows). In the unfiltered *x–z* section through these markers, the streaks produced by the side rays of aren’t as spurious as in the reconstruction of the cerebellum molecular layer (Fig.7D, bottom). However, the angular filter suppressed the side rays (white arrows) and decreased intensity in the side-maxima (black arrows). On the other hand, the top and bottom boundaries of the balls are smeared after angular filtering due to a loss of the *z*-resolutions (Fig.7E, white arrowheads) caused by filtering with an angular filter with a tight central stripe (*bfly24-4-0.2-8-4-4*).

## 5 Discussion

In this study, we have demonstrated that the proposed angular filtering mitigates one of the major noise sources in single-axis electron tomographic reconstructions, which is indicated by results of numerical analysis (Table 2, supp. Tables 1–3) as well as by the suppression of background clutter in cross-sections through reconstructions of real specimens (Figs. 4–7 and Movies 1–5).

The drawback of the proposed filter is a further loss of z-resolution in the reconstructed volumes due to higher intensities of its impulse response *W_A_*(*x*,*z*) along the *z*-axis (Fig.2D). This unwanted effect is apparent particularly in enlarged images of reconstructed details and in the images of intensity differences between filtered and unfiltered version of the same *x*–*z* cross-section (Fig.4H and Fig.7D–F), as well as in Mov. 1–3. However, if a filter with a sufficiently large central stripe is used for filtering of reconstructions computed from projections with low signal-to-noise ratios, the decrease of reconstruction quality due to the loss of resolution in *z*-direction is compensated by the reduction of the ray artefacts, which is documented by the numerical results presented in Table 2 and supp. Tables 1–3. In addition, angular filters well handle the most spurious streaks arising from features with extreme intensities, such as colloidal gold markers (Fig.7).

Naturally, since angular filtering is basically a directional filtering specifically aimed at the suppression of the side rays of the single-axis tilting tomographic impulse response *W*_2_(*x*,*z*), it cannot substitute any kind of standard filters used in tomography, like the omnidirectional low-pass filters, median filters or the NAD filters, but it provides a valuable complement to them. In our cryo-ET examples (Figs. 5 and 6; Movies 4 and 5) we performed angular filtering prior to NAD filtering because NAD relies on the detection of local intensity gradients (Fernandez, 2009; Frangakis and Hegerl, 2001, 2006), which are likely to be more accurately estimated after the removal of the side rays.

Since angular filters act in the Fourier domain, they can be easily combined either with low-pass filters, which can be used to create a filter capable of suppression of two common electron tomographic noise sources in a single step, or with the weighted back-projection R-filters, which would allow a seamless incorporation of angular filters into existing tomographic reconstruction workflows.

Angular filters also act as mask filters zeroing out the non-tomographic noise components in the missing wedge, a technique used e.g. in (Lanzavecchia and Bellon, 1996). The discrete design of angular filters allows their modifications that would protect arbitrary spatial frequencies in the missing wedge, at which the Fourier transform values could be estimated by interpolation or by regularization techniques. Unlike the regularization methods however, angular filtering does not rely on any assumptions about the composition of specimens, their spatial distribution or about the properties of noise in projections.

The proposed angular filter is intended for use with single-axis cryo-electron tomograms, but it can be modified to fit other tomographic data collection schemes with different geometries of the area of missing data. In the double-axis tilting geometry with two orthogonal tilting axes, the width of the central stripe may increase with increasing spatial frequency along the tilting axis in order to protect the second-axis tilt series data. In the random-conical tilt geometries, the 2D single-axis angular filter can be rotated around the *Z* axis to obtain the intended data area with smooth transition towards the missing cone region. The suppression of signal at middle and high frequencies in the high-tilt projections may also be useful if there are doubts about quality of high-tilt projections where the projection condition (Hawkes, 2006) may be violated.

## 6 Conclusions

Angular filtering is a novel method for suppression of the missing wedge ray artefacts in electron tomographic reconstructions. The acquired results, both numerical and visual, indicate that angular filtering in connection with three of the standard reconstruction methods (WBP, DFM, SIRT) can reduce tomographic noise at the single-axis tilting acquisition scheme within a limited tilting range. This simplifies interpretation and segmentation of electron tomographic reconstructions where sub-tomogram averaging is not possible, especially at low signal-to-noise ratios in acquired projections. A Matlab application for angular filtering of both single-axis tomographic reconstructions and sets of aligned projections named BflyTool is available for download at http://lge.lf1.cuni.cz/software.php.

## Figures and Tables

**Table 1:** Notation, parameters and smoothing ability of angular filters used in this study. Variances in the last column were computed from an annular region *AR*(*x*,*z*) (inner radius: 2 pixels, outer radius: 25 pixels, centered at *x* = 0, *z* = 0) extracted from the numerically computed impulse responses *W_A_*(*x*,*z*) of the angular filters and *W*_2_(*x*,*z*) of the missing wedge, respectively.

Filter notation	Missing wedge ramp			Central stripe ramp			Background smoothing ability *r_A_*
	Length (pixels)	Order	Highest tilt weight	Length (pixels)	Order	Cutoff (pixels)	$\frac{\sigma^{2}(W_{A}(\mathrm{AR}(x\text{,}z)))}{\sigma^{2}(W_{2}(\mathrm{AR}(x\text{,}z)))}$
*bfly20-4-0.5-15-4-10*	20	4	0.5	15	4	10	0.85
*bfly20-4-0.2-15-4-10*	20	4	0.2	15	4	10	0.80
*bfly20-4-0.13-10*	20	4	0.13	15	4	10	0.78
*bfly20-4-0.2-25-4-20*	20	4	0.2	25	4	20	0.79
*bfly20-4-0.2-8-2-4*	20	4	0.2	8	2	4	0.76
*bfly10-4-0.2-15-4-10*	10	4	0.2	15	4	10	0.86
*bfly40-4-0.2-15-4-10*	40	4	0.2	15	4	10	0.69

**Table 2:** Performance of angular filtering of the random-cylinders test volumes expressed by mean relevancies of improvement of FOMs. In case of the foreground mean separability FOMs and SNR in reconstructions, simple ratio of angularly filtered versus unfiltered FOMs or SNRs, respectively, was used. Statistically significant improvement is depicted by numbers in bold, inferior performance by standard font, and insignificant changes in performance by underlined italic font. Filters in rows 1,2 and 3 demonstrate the effect of a decreasing weight of the missing wedge ramp at the highest-tilt projection, filters in rows 4, 2 and 5 the effect of narrowing of the central stripe, and filters in rows 6, 2 and 7 the effect of an increasing length of the missing wedge ramp.

	Angular filter	Whole volume eFOM						Signal voxels eFOM							Background voxels eFOM				
		SNR in projections						SNR in projections							SNR in projections				

0.01	0.1	0.5	1	5	∞	0.01	0.1	0.5	1	5	∞	0.01	0.1	0.5	1	5	∞

1	*bfly20-4-0.5-15-4-10*	**5.01**	**2.93**	**2.85**	**2.71**	−0.04	−2.38	**3.29**	**2.82**	**0.52**	−2.59	−6.18	−6.35	**5.06**	**2.93**	**2.91**	**2.98**	**3.04**	**3.46**
2	*bfly20-4-0.2-15-4-10*	**7.85**	**7.84**	**7.64**	**7.34**	**1.05**	**−**4.38	**7.89**	**7.63**	**3.09**	−2.90	−9.80	−10.25	**7.85**	**7.84**	**7.77**	**7.86**	**6.49**	**4.25**
3	*bfly20-4-0.13-15-4-10*	**11.08**	**11.04**	**10.79**	**10.36**	**1.95**	**−**5.27	**11.10**	**10.79**	**5.01**	−2.56	−11.31	−11.81	**11.08**	**11.04**	**10.94**	**11.02**	**8.59**	**4.31**
4	*bfly20-4-0.2-25-4-20*	**7.42**	**7.41**	**7.26**	**7.10**	**2.77**	−0.83	**7.45**	**7.32**	**4.81**	**1.62**	−2.04	−2.22	**7.41**	**7.41**	**7.32**	**7.38**	**5.18**	**1.21**
5	*bfly20-4-0.2-8-2-4*	**8.16**	**8.13**	**7.74**	**6.80**	−8.33	−21.50	**8.19**	**7.45**	−6.84	−25.36	−47.77	−47.99	**8.16**	**8.15**	**8.13**	**8.45**	**11.57**	**17.83**
6	*bfly10-4-0.2-15-4-10*	**4.48**	**4.48**	**4.36**	**4.17**	0.08	−3.45	**4.51**	**4.35**	**1.22**	−2.96	−7.72	−8.04	**4.48**	**4.48**	**4.44**	**4.53**	**3.99**	**3.29**
7	*bfly40-4-0.2-15-4-10*	**15.54**	**15.43**	**15.12**	**14.53**	**3.68**	−5.64	**15.54**	**15.16**	**7.98**	−1.47	−12.32	−12.87	**15.54**	**15.44**	**15.32**	**15.34**	**11.69**	**4.96**

		Range FOM						Signal voxels std. deviation FOM						Background voxels std. deviation FOM					
		SNR in projections						SNR in projections						SNR in projections					
		0.01	0.1	0.5	1	5	∞	0.01	0.1	0.5	1	5	∞	0.01	0.1	0.5	1	5	∞

1	*bfly20-4-0.5-15-4-10*	**2.12**	**1.76**	**1.56**	**2.16**	**5.09**	**4.06**	**1.74**	**1.48**	**1.48**	**1.55**	**1.70**	**2.13**	**2.56**	**1.48**	**1.47**	**1.51**	**1.55**	**1.80**
2	*bfly20-4-0.2-15-4-10*	**4.83**	**4.35**	**4.74**	**5.77**	**9.74**	**8.47**	**4.05**	**4.00**	**3.93**	**3.90**	**2.88**	**3.27**	**4.01**	**4.00**	**3.96**	**4.01**	**3.33**	**2.24**
3	*bfly20-4-0.13-15-4-10*	**6.70**	**5.90**	**6.81**	**7.52**	**11.11**	**10.04**	**5.74**	**5.69**	**5.55**	**5.38**	**3.26**	**3.49**	**5.70**	**5.68**	**5.63**	**5.67**	**4.43**	**2.28**
4	*bfly20-4-0.2-25-4-20*	**4.70**	**4.22**	**4.61**	**5.07**	*3.03*	**3.50**	**3.82**	**3.78**	**3.62**	**3.34**	**0.94**	**0.32**	**3.78**	**3.78**	**3.73**	**3.76**	**2.64**	**0.63**
5	*bfly20-4-0.2-8-2-4*	**4.79**	**4.27**	**5.28**	**7.84**	**21.97**	*6.71*	**4.21**	**4.16**	**4.41**	**5.22**	**10.11**	**12.54**	**4.17**	**4.16**	**4.16**	**4.33**	**6.12**	**9.83**
6	*bfly10-4-0.2-15-4-10*	**2.82**	**2.82**	**2.63**	**3.56**	**5.65**	**6.11**	**2.29**	**2.27**	**2.23**	**2.24**	**1.71**	**2.09**	**2.27**	**2.27**	**2.25**	**2.29**	**2.04**	**1.73**
7	*bfly40-4-0.2-15-4-10*	**8.33**	**8.20**	**8.52**	**9.37**	**14.74**	**12.02**	**8.13**	**8.07**	**7.88**	**7.48**	**4.15**	**4.19**	**8.10**	**8.04**	**7.98**	**7.99**	**6.07**	**2.62**

		Foreground mean separability FOM						Detectability error FOM						SNR					
		SNR in projections						SNR in projections						SNR in projections					
		**0.01**	**0.1**	**0.5**	**1**	**5**	∞	0.01	0.1	0.5	1	5	∞	0.01	0.1	0.5	1	5	∞

1	*bfly20-4-0.5-15-4-10*	*1.07*	0.98	0.99	0.99	0.99	0.99	*0.00*	*0.00*	0.00	−0.19	−6.88	−8.89	*1.03*	*1.00*	1.00	1.00	**1.00**	**1.01**
2	*bfly20-4-0.2-15-4-10*	*0.87*	*1.00*	0.99	0.99	0.98	0.99	*0.00*	*0.00*	0.00	−0.07	−7.69	−17.44	**1.04**	**1.04**	**1.04**	**1.04**	**1.02**	1.00
3	*bfly20-4-0.13-15-4-10*	*1.00*	*1.00*	*1.00*	*1.00*	0.98	0.98	*0.00*	*0.00*	*0.00*	**0.10**	−7.06	−22.72	**1.08**	**1.07**	**1.07**	**1.07**	**1.04**	0.99
4	*bfly20-4-0.2-25-4-20*	*1.09*	**1.04**	**1.03**	**1.02**	*1.00*	0.99	*0.00*	*0.00*	**0.00**	**0.47**	**4.72**	−4.81	**1.08**	**1.07**	**1.07**	**1.07**	**1.04**	**1.00**
5	*bfly20-4-0.2-8-2-4*	*0.87*	0.84	0.84	0.84	0.89	0.91	*0.00*	**0.00**	0.00	−1.53	−91.55	−121.27	0.87	0.88	0.88	0.88	0.91	**0.99**
6	*bfly10-4-0.2-15-4-10*	*1.01*	*0.99*	0.99	0.99	0.98	0.98	*0.00*	*0.00*	0.00	−0.17	−8.97	−16.13	**1.02**	**1.01**	**1.01**	**1.01**	**1.01**	**1.00**
7	*bfly40-4-0.2-15-4-10*	*1.08*	*1.02*	**1.02**	**1.02**	0.98	0.98	*0.00*	*0.00*	**0.00**	**0.42**	−2.66	−22.20	**1.13**	**1.12**	**1.11**	**1.11**	**1.07**	0.99

## Appendix A Supplementary data

Supplementary data 1 This document file contains Supplementary Movie 1.

Supplementary data 2 This document file contains Supplementary Movie 2.

Supplementary data 3 This document file contains Supplementary Movie 3.

Supplementary data 4 This document file contains Supplementary Movie 4.

Supplementary data 5 This document file contains Supplementary Movie 5.

Supplementary data 6 This document file contains Supplementary Table 1.

Supplementary data 7 This document file contains Supplementary Table 2.

Supplementary data 8 This document file contains Supplementary Table 3.
