# Corrigendum to “A simple Fourier filter for suppression of the missing wedge ray artefacts in single-axis electron tomographic reconstructions” [J. Struct. Biol. 186(1) (2014) 141–152]

**DOI:** 10.1016/j.jsb.2014.05.008

**Published:** 2014-07

**Authors:** Lubomír Kováčik, Sami Kereïche, Johanna L. Höög, Pavel Jůda, Pavel Matula, Ivan Raška

**Affiliations:** a Charles University in Prague, First Faculty of Medicine, Institute of Cellular Biology and Pathology, Albertov 4, 128 01 Prague 2, Czech Republic; b Sir William Dunn School of Pathology, University of Oxford, South Parks Road, Oxford OX1 3RE, UK; c MPI-CBG, Photenhauerstr. 108, 01307 Dresden, Germany; d Centre for Biomedical Image Analysis, Faculty of Informatics, Masaryk University, Brno, Czech Republic

The name of Sami Kereïche appeared incorrectly. The corrected author line appears above.

