# Supplementary material for: A simple Fourier filter for suppression of the missing wedge ray artefacts in single-axis electron tomographic reconstructions
Source: J Struct Biol. 2014 Apr;186(1):141–52. doi: 10.1016/j.jsb.2014.02.004 (PMC3991334; doi:10.1016/j.jsb.2014.02.004)
Supplement: Supplementary data 1 — This document file contains Supplementary Movie 1. [file mmc1.pptx]

## Slide 1
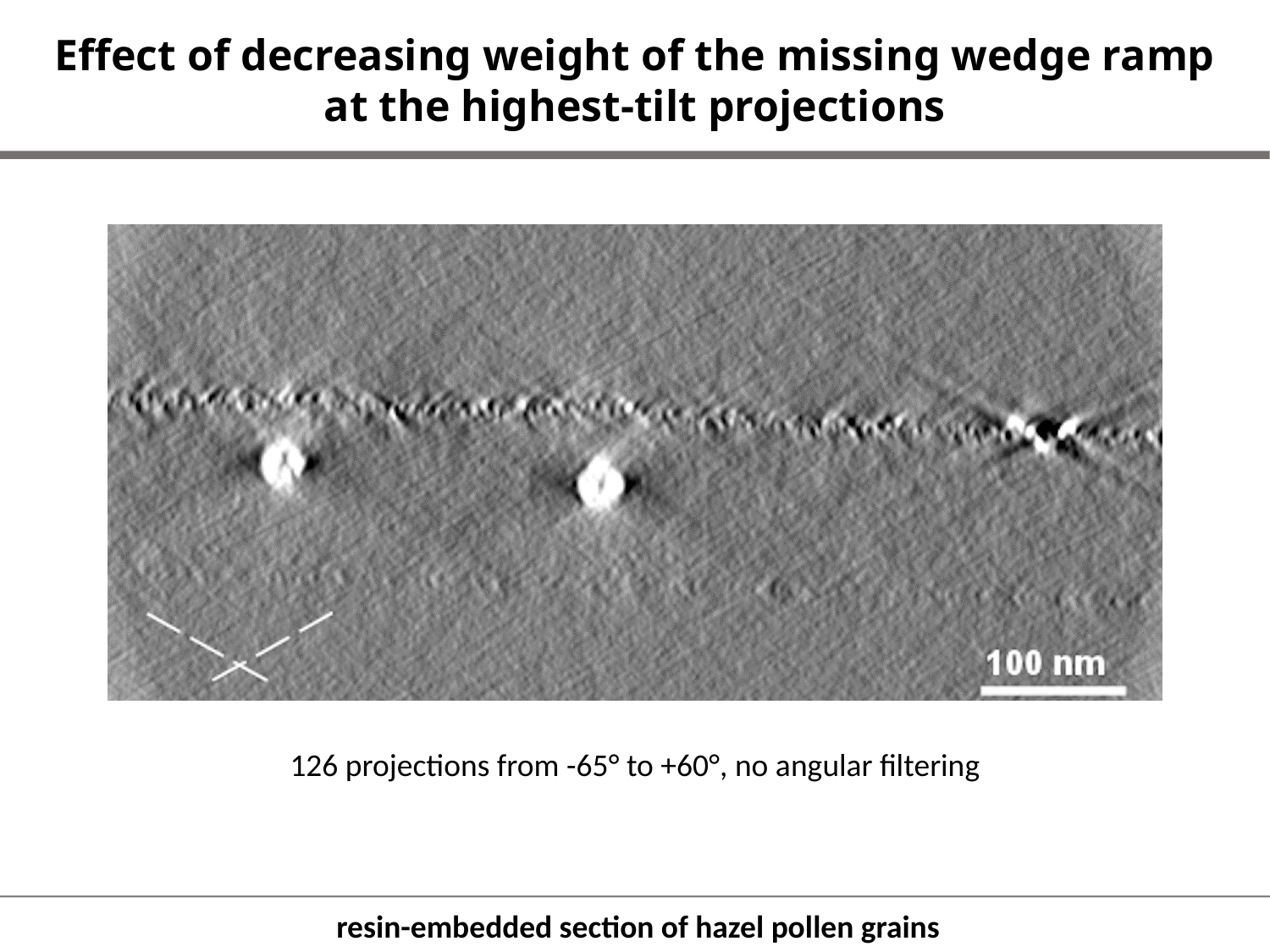

Effect of decreasing weight of the missing wedge ramp at the highest-tilt projections
126 projections from -65° to +60°, no angular filtering
 resin-embedded section of hazel pollen grains

## Slide 2
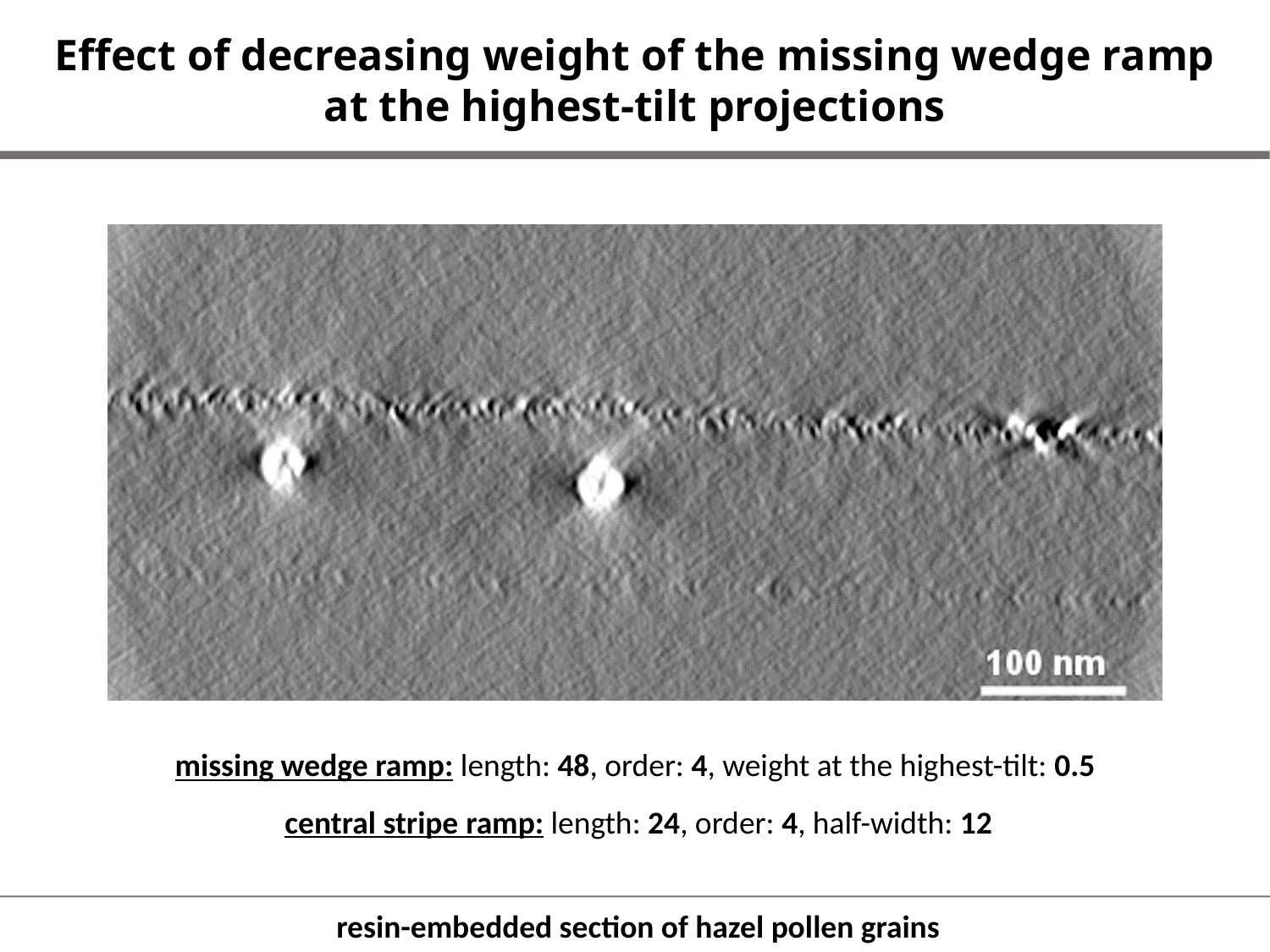

Effect of decreasing weight of the missing wedge ramp at the highest-tilt projections
missing wedge ramp: length: 48, order: 4, weight at the highest-tilt: 0.5
 central stripe ramp: length: 24, order: 4, half-width: 12
 resin-embedded section of hazel pollen grains

## Slide 3
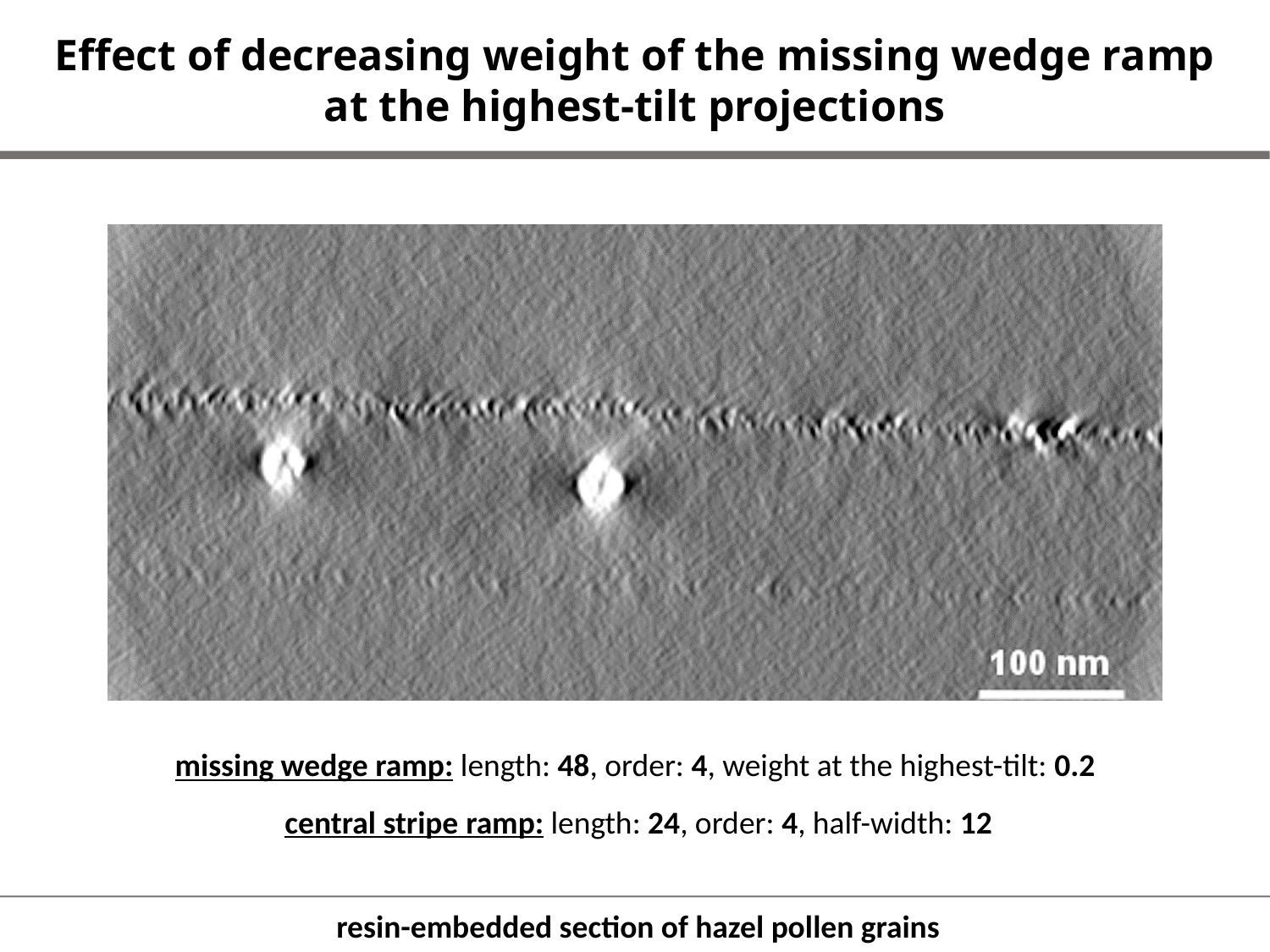

Effect of decreasing weight of the missing wedge ramp at the highest-tilt projections
missing wedge ramp: length: 48, order: 4, weight at the highest-tilt: 0.2
 central stripe ramp: length: 24, order: 4, half-width: 12
 resin-embedded section of hazel pollen grains

## Slide 4
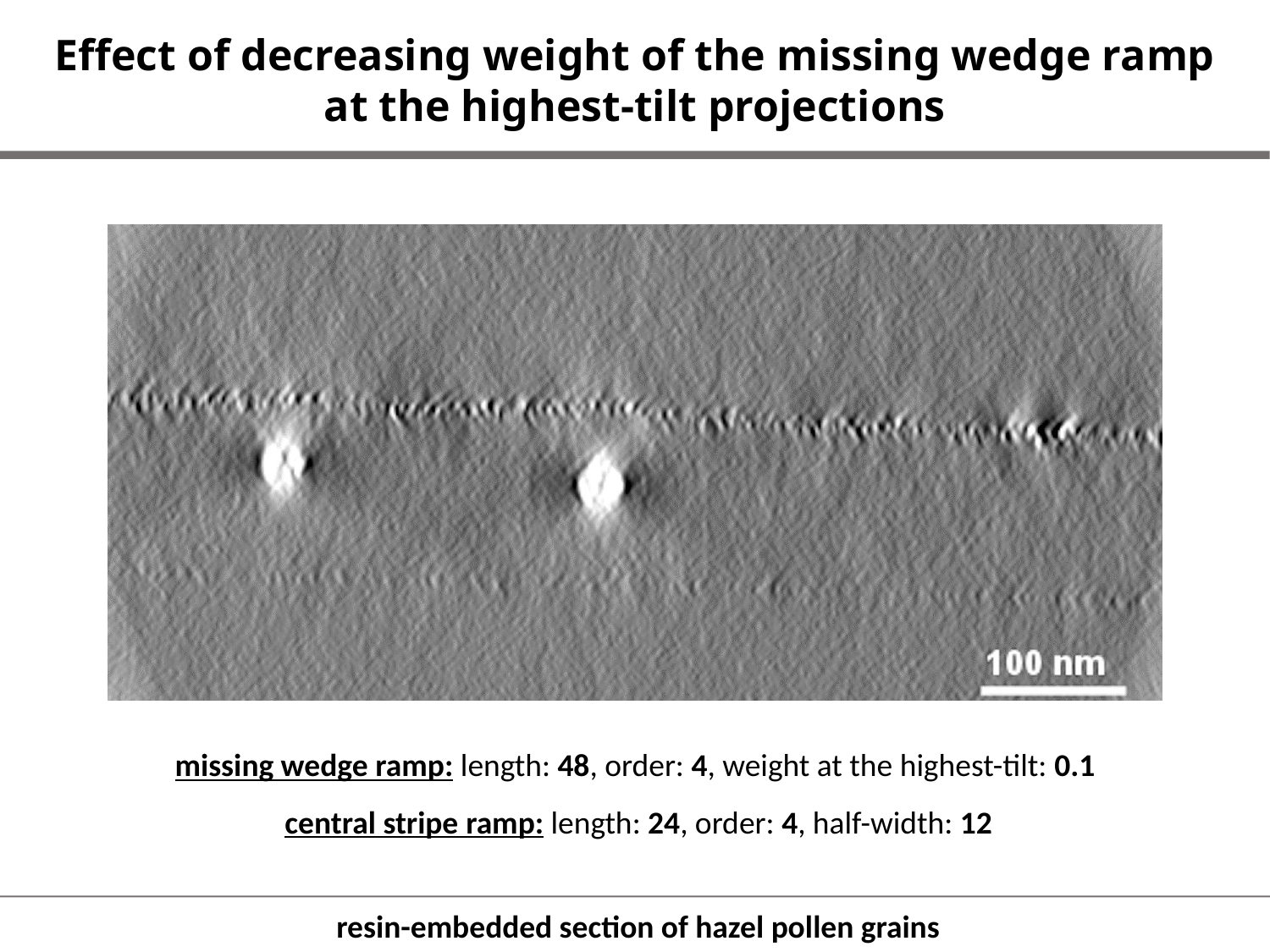

Effect of decreasing weight of the missing wedge ramp at the highest-tilt projections
missing wedge ramp: length: 48, order: 4, weight at the highest-tilt: 0.1
 central stripe ramp: length: 24, order: 4, half-width: 12
 resin-embedded section of hazel pollen grains

## Slide 5
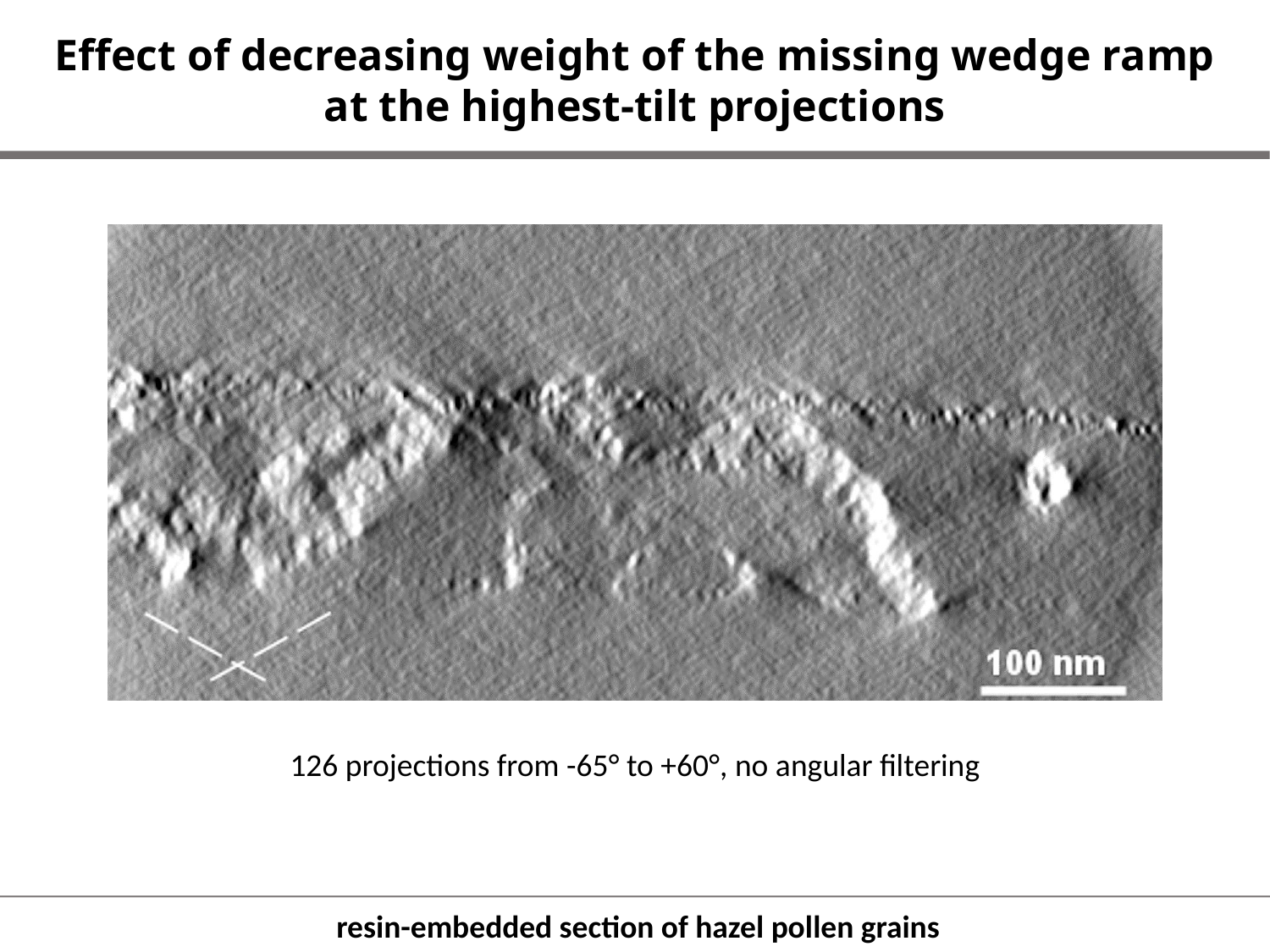

Effect of decreasing weight of the missing wedge ramp at the highest-tilt projections
126 projections from -65° to +60°, no angular filtering
 resin-embedded section of hazel pollen grains

## Slide 6
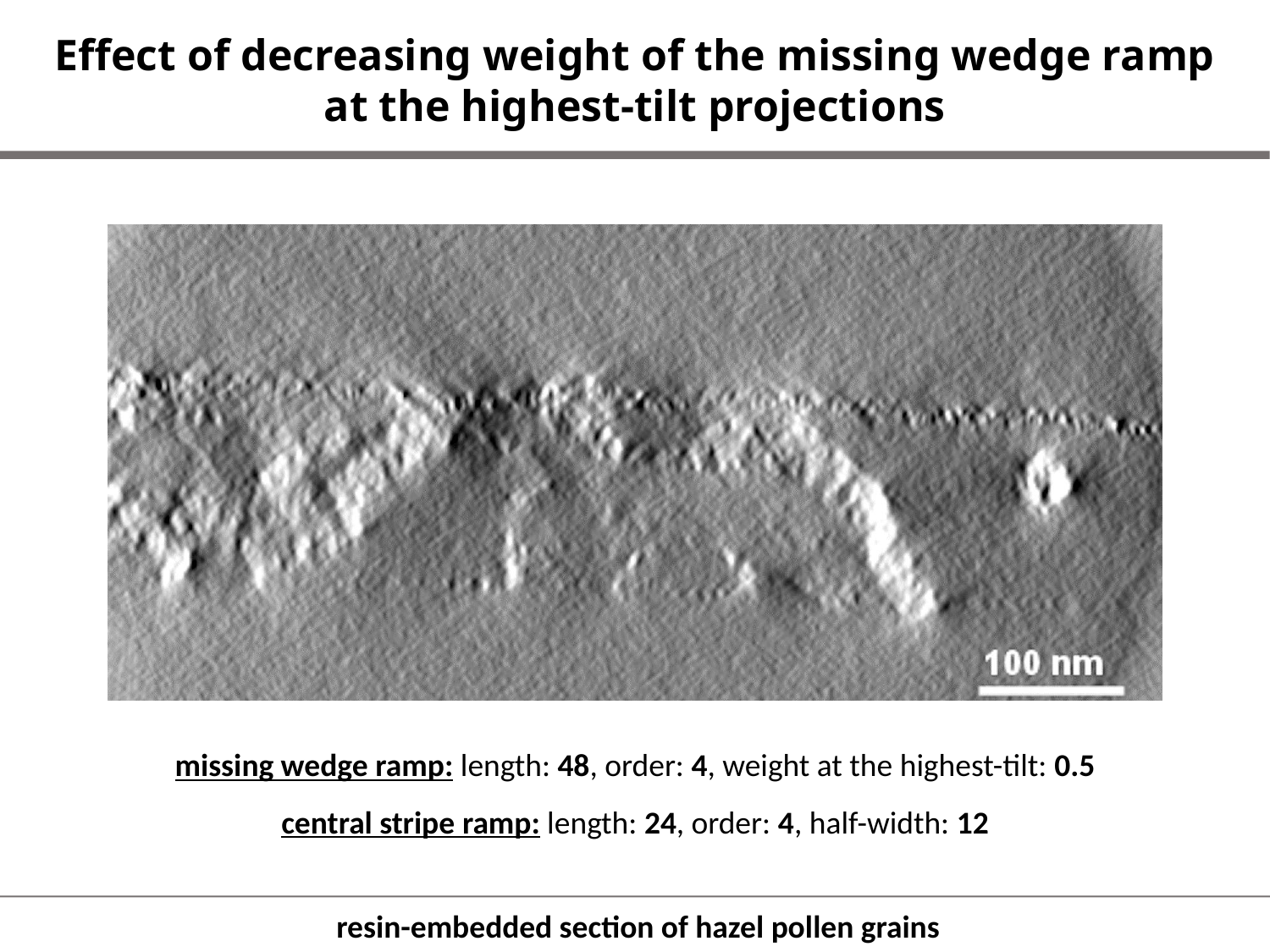

Effect of decreasing weight of the missing wedge ramp at the highest-tilt projections
missing wedge ramp: length: 48, order: 4, weight at the highest-tilt: 0.5
 central stripe ramp: length: 24, order: 4, half-width: 12
 resin-embedded section of hazel pollen grains

## Slide 7
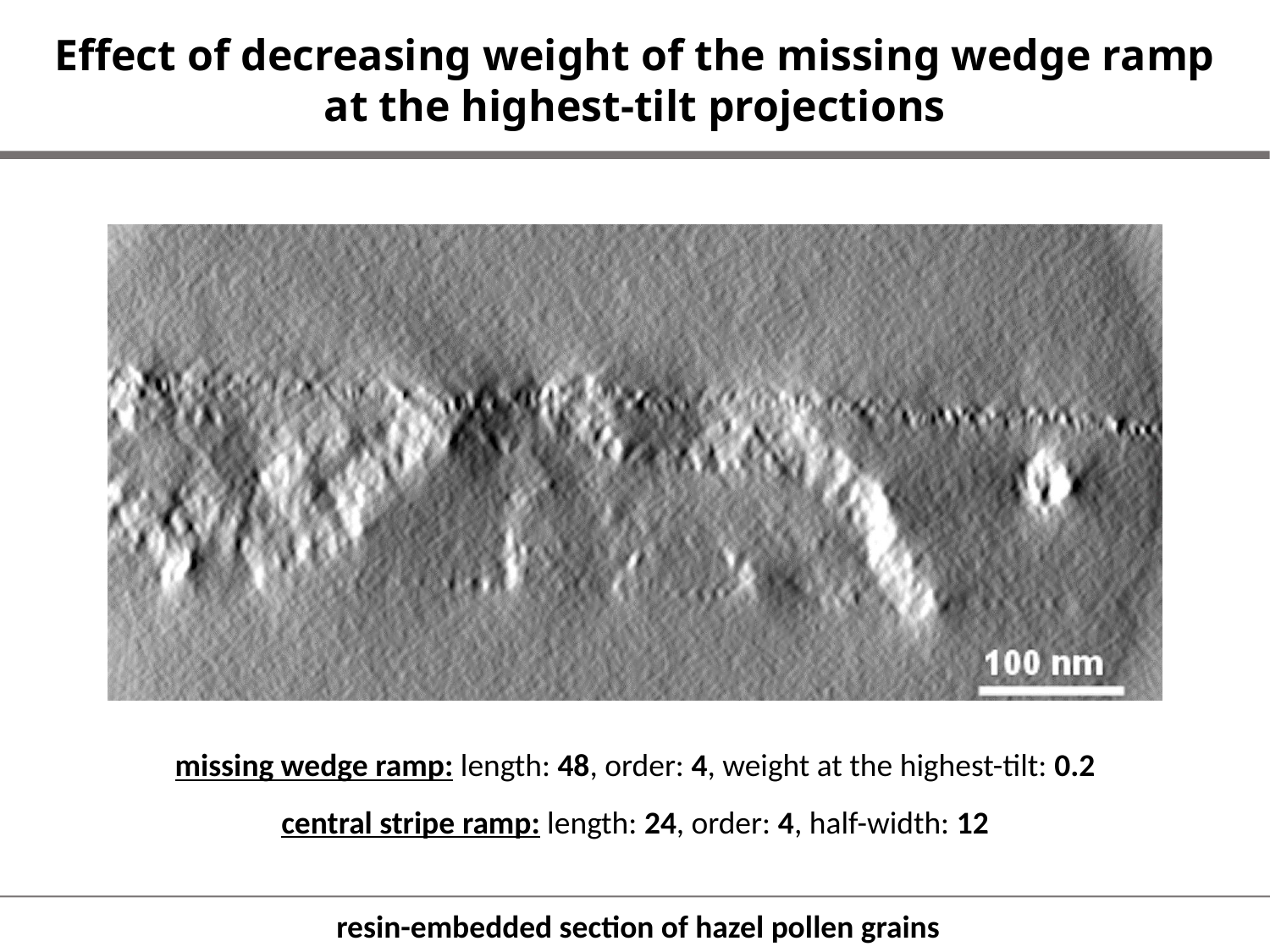

Effect of decreasing weight of the missing wedge ramp at the highest-tilt projections
missing wedge ramp: length: 48, order: 4, weight at the highest-tilt: 0.2
 central stripe ramp: length: 24, order: 4, half-width: 12
 resin-embedded section of hazel pollen grains

## Slide 8
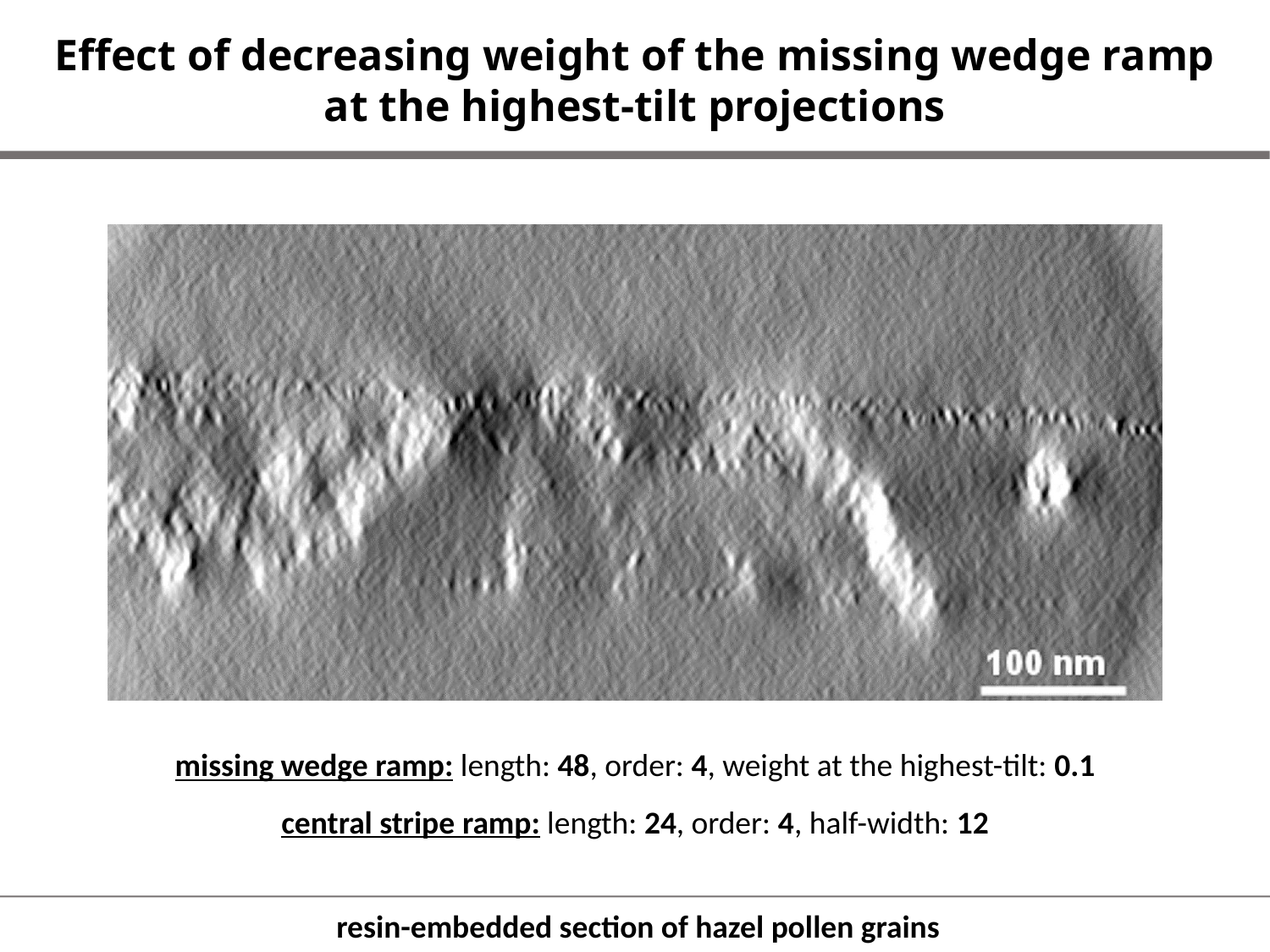

Effect of decreasing weight of the missing wedge ramp at the highest-tilt projections
missing wedge ramp: length: 48, order: 4, weight at the highest-tilt: 0.1
 central stripe ramp: length: 24, order: 4, half-width: 12
 resin-embedded section of hazel pollen grains

## Slide 9
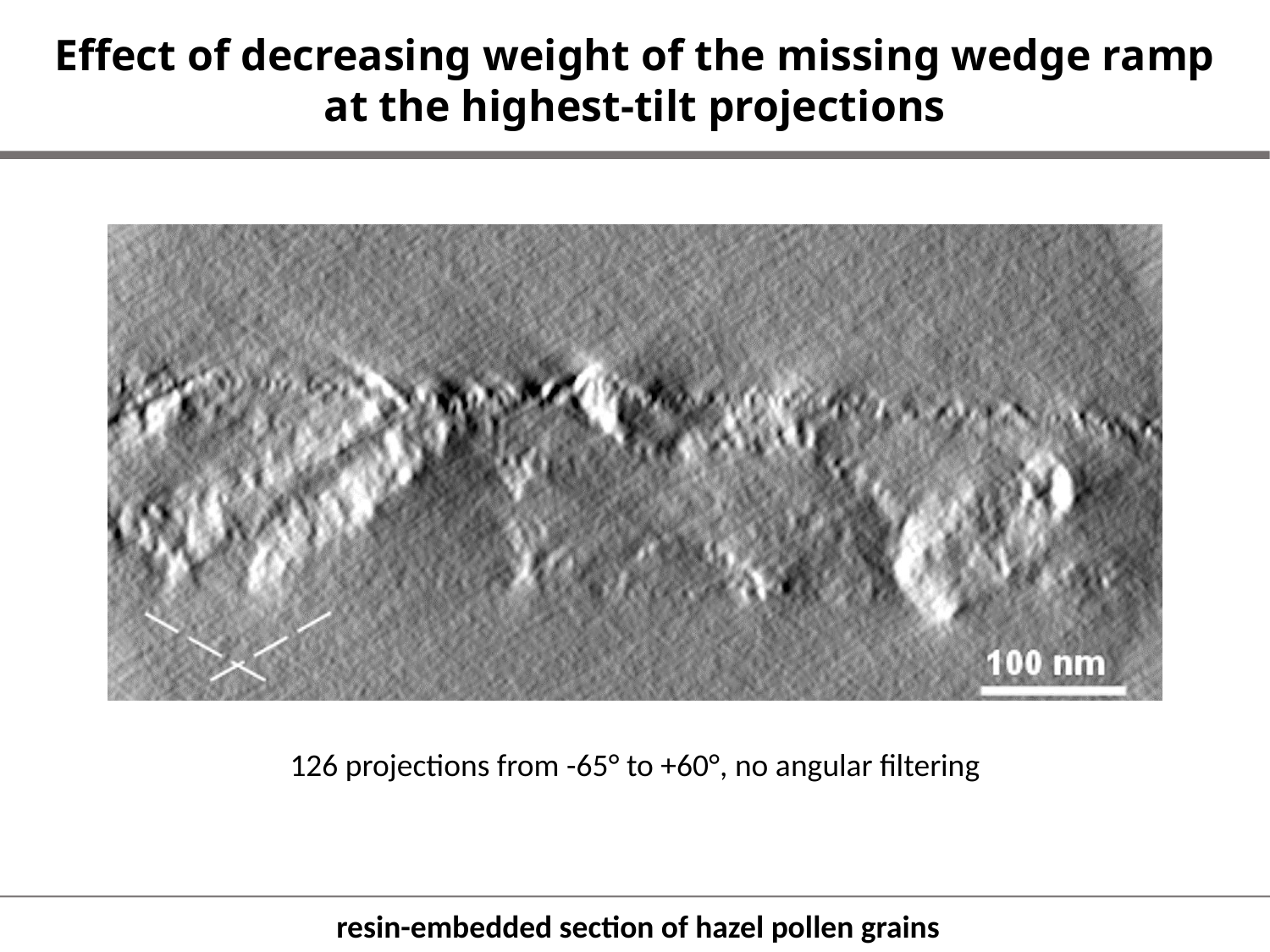

Effect of decreasing weight of the missing wedge ramp at the highest-tilt projections
126 projections from -65° to +60°, no angular filtering
 resin-embedded section of hazel pollen grains

## Slide 10
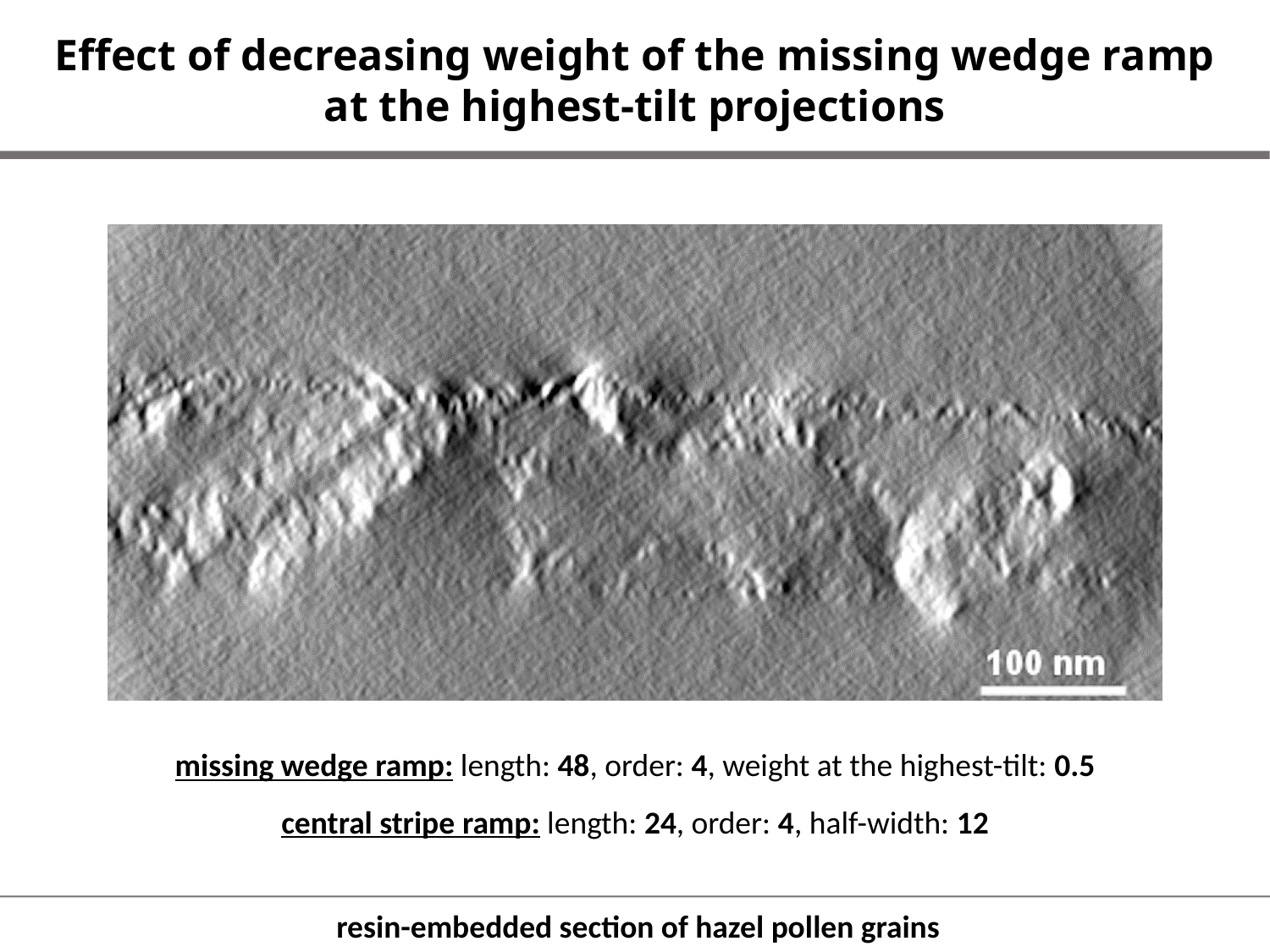

Effect of decreasing weight of the missing wedge ramp at the highest-tilt projections
missing wedge ramp: length: 48, order: 4, weight at the highest-tilt: 0.5
 central stripe ramp: length: 24, order: 4, half-width: 12
 resin-embedded section of hazel pollen grains

## Slide 11
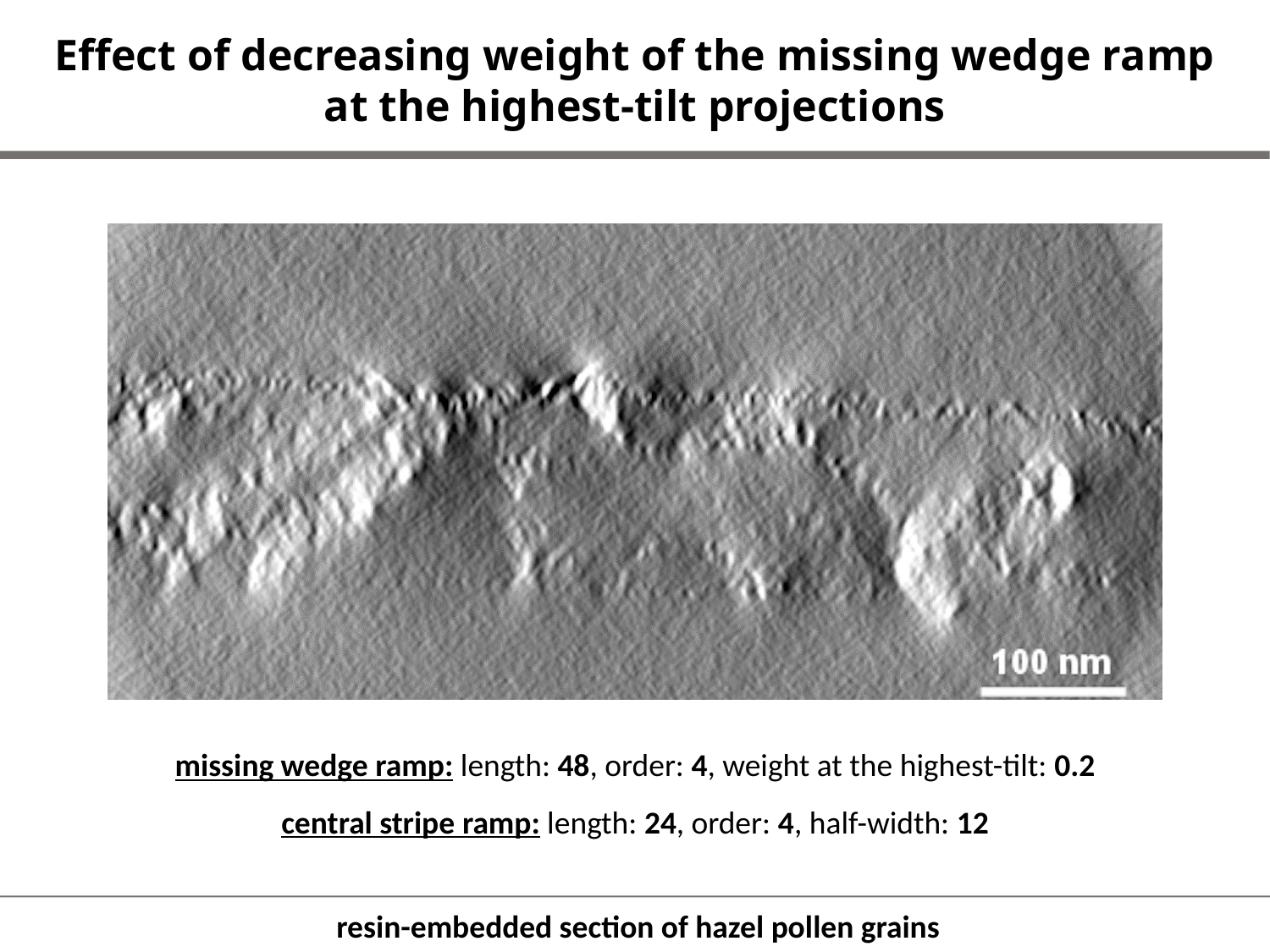

Effect of decreasing weight of the missing wedge ramp at the highest-tilt projections
missing wedge ramp: length: 48, order: 4, weight at the highest-tilt: 0.2
 central stripe ramp: length: 24, order: 4, half-width: 12
 resin-embedded section of hazel pollen grains

## Slide 12
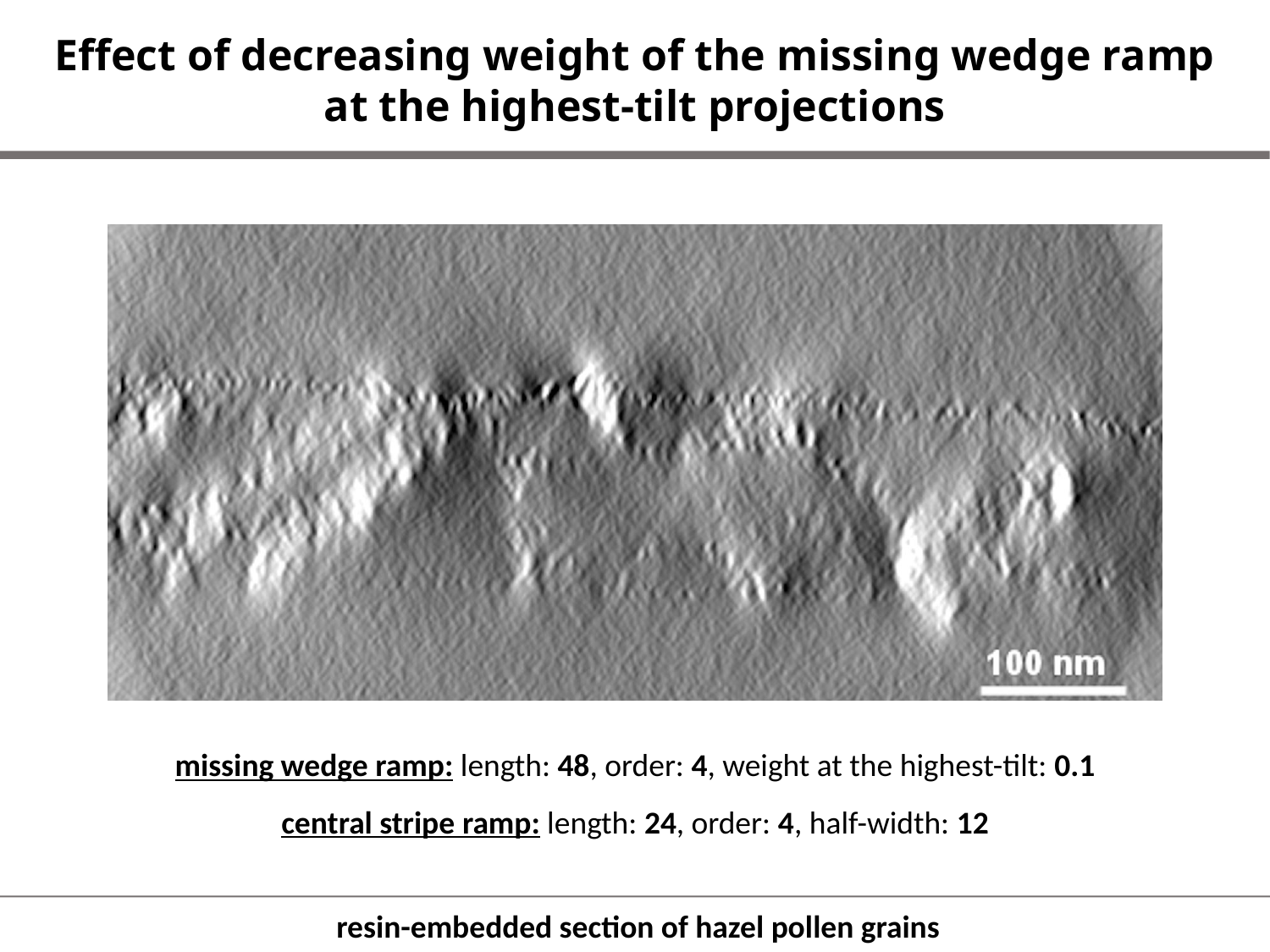

Effect of decreasing weight of the missing wedge ramp at the highest-tilt projections
missing wedge ramp: length: 48, order: 4, weight at the highest-tilt: 0.1
 central stripe ramp: length: 24, order: 4, half-width: 12
 resin-embedded section of hazel pollen grains
