# Supplementary material for: A simple Fourier filter for suppression of the missing wedge ray artefacts in single-axis electron tomographic reconstructions
Source: J Struct Biol. 2014 Apr;186(1):141–52. doi: 10.1016/j.jsb.2014.02.004 (PMC3991334; doi:10.1016/j.jsb.2014.02.004)
Supplement: Supplementary data 3 — This document file contains Supplementary Movie 3. [file mmc3.pptx]

## Slide 1
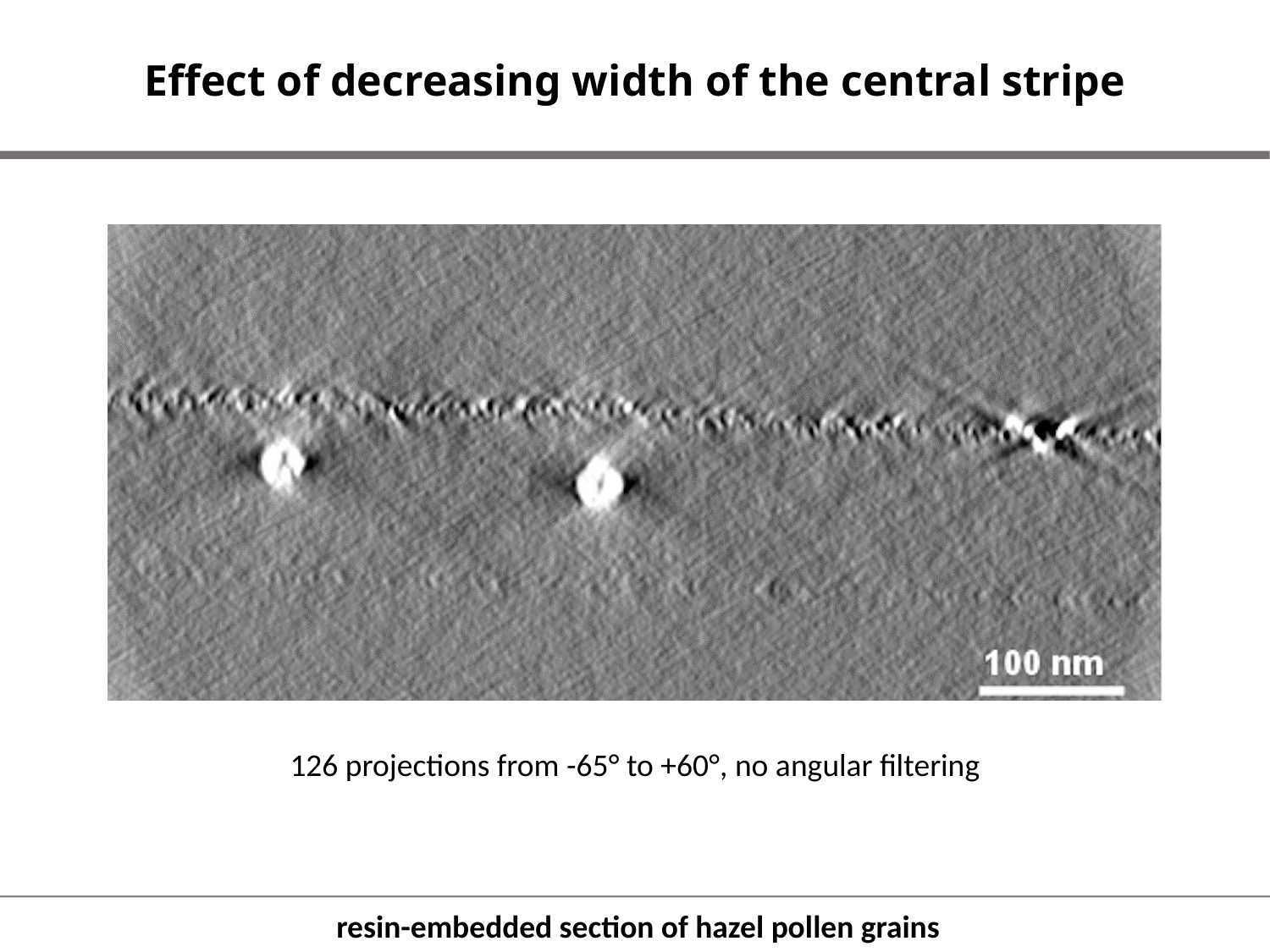

Effect of decreasing width of the central stripe
126 projections from -65° to +60°, no angular filtering
 resin-embedded section of hazel pollen grains

## Slide 2
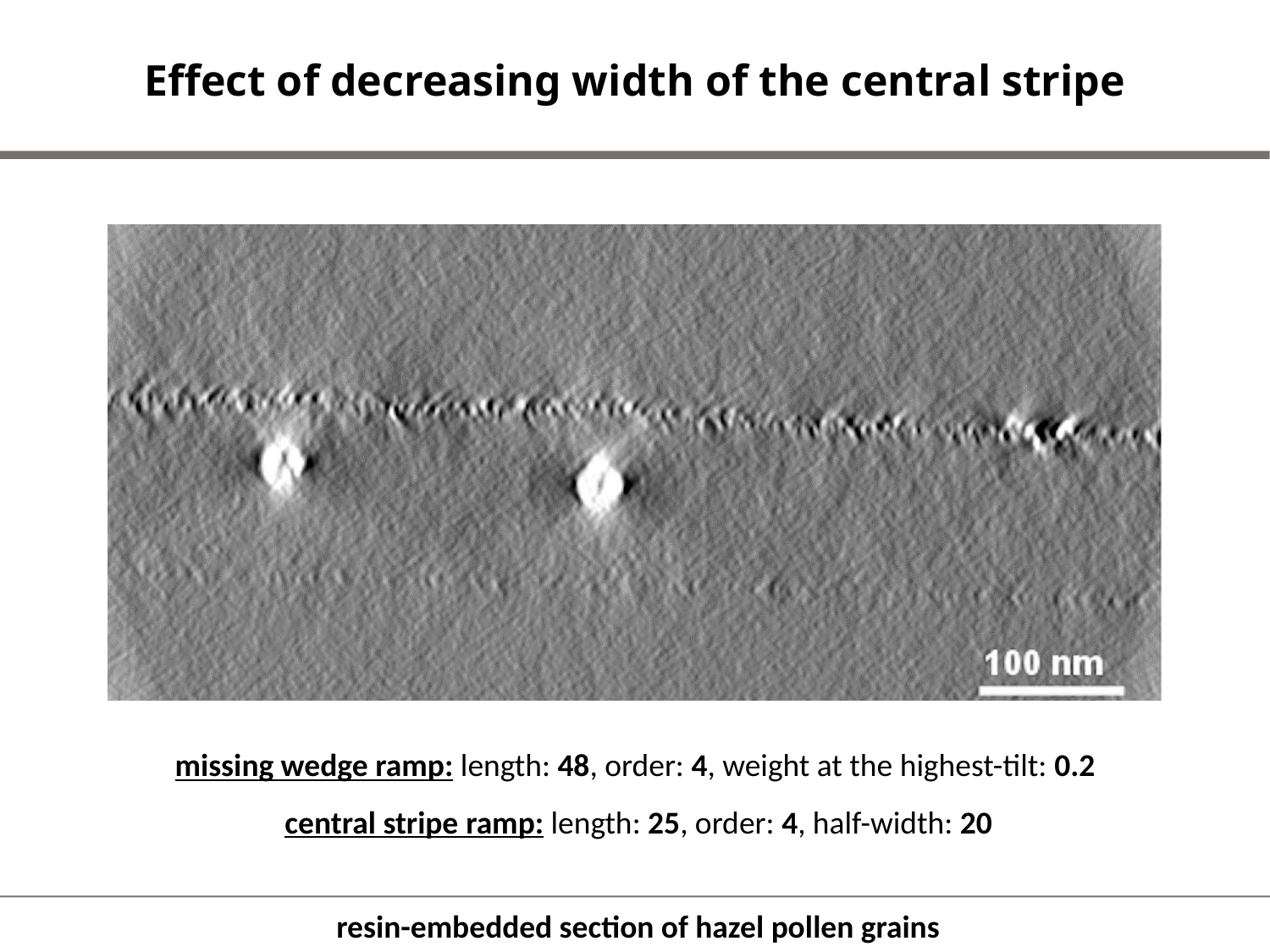

Effect of decreasing width of the central stripe
missing wedge ramp: length: 48, order: 4, weight at the highest-tilt: 0.2
 central stripe ramp: length: 25, order: 4, half-width: 20
 resin-embedded section of hazel pollen grains

## Slide 3
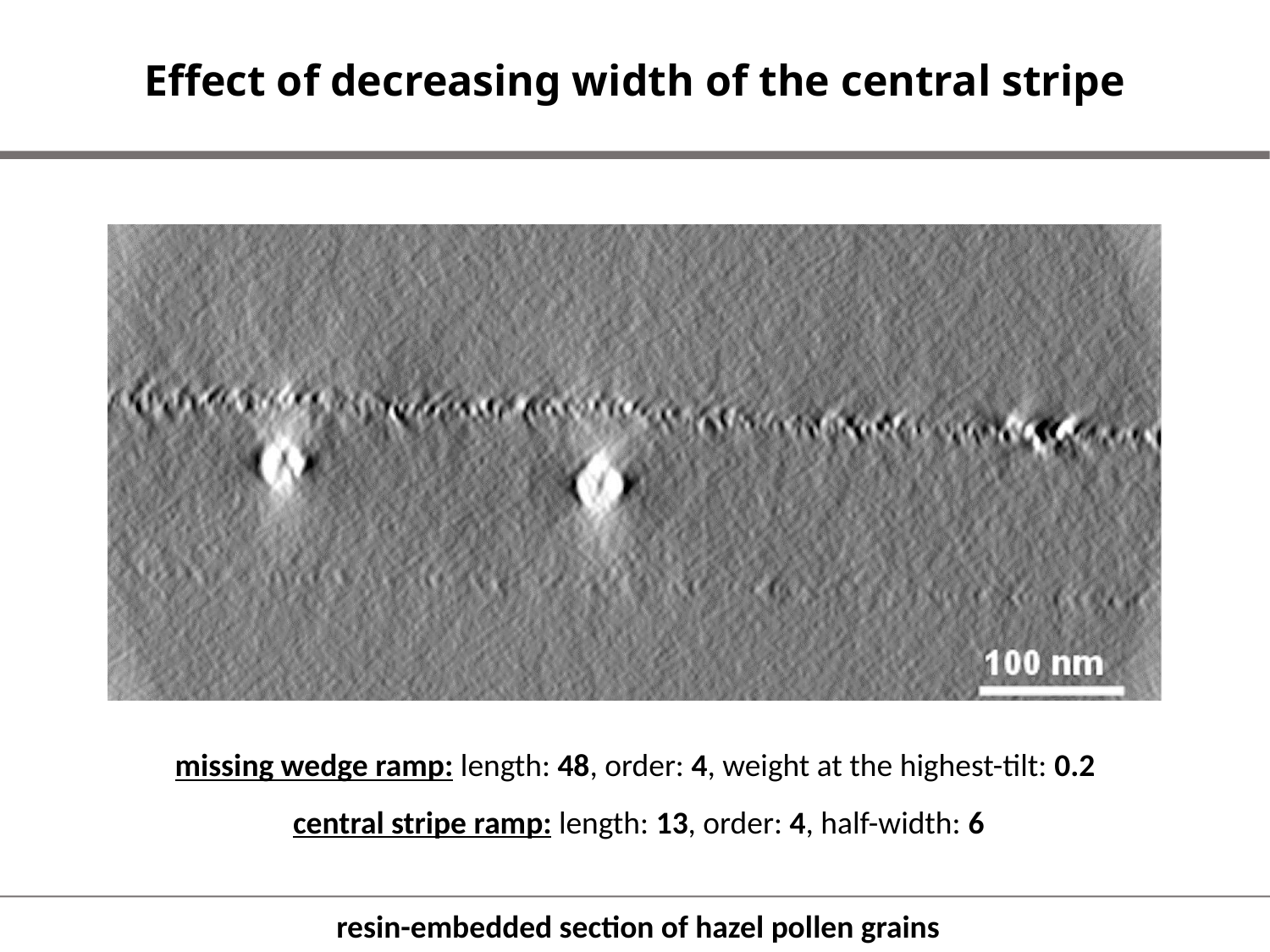

Effect of decreasing width of the central stripe
missing wedge ramp: length: 48, order: 4, weight at the highest-tilt: 0.2
 central stripe ramp: length: 13, order: 4, half-width: 6
 resin-embedded section of hazel pollen grains

## Slide 4
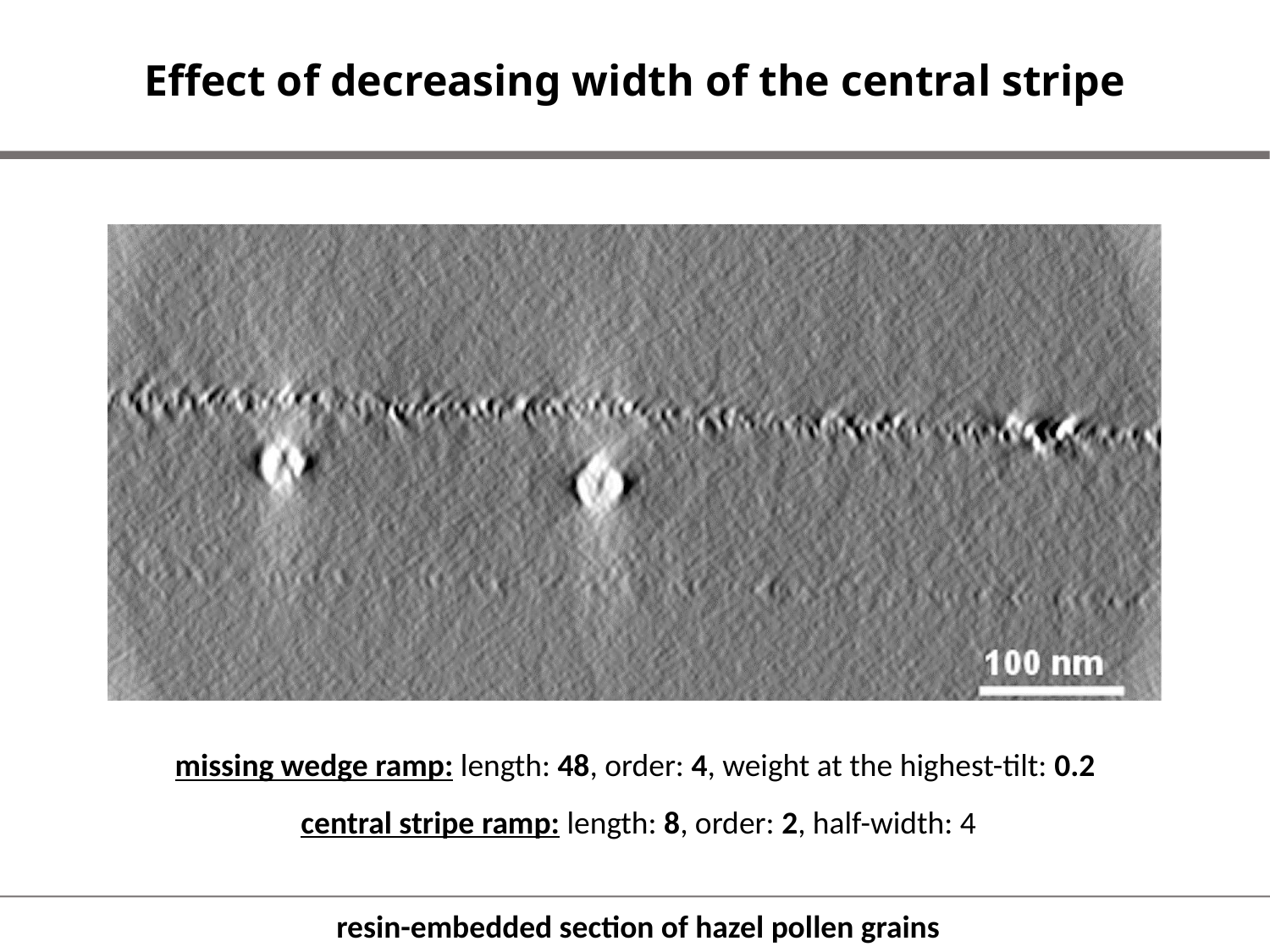

Effect of decreasing width of the central stripe
missing wedge ramp: length: 48, order: 4, weight at the highest-tilt: 0.2
 central stripe ramp: length: 8, order: 2, half-width: 4
 resin-embedded section of hazel pollen grains

## Slide 5
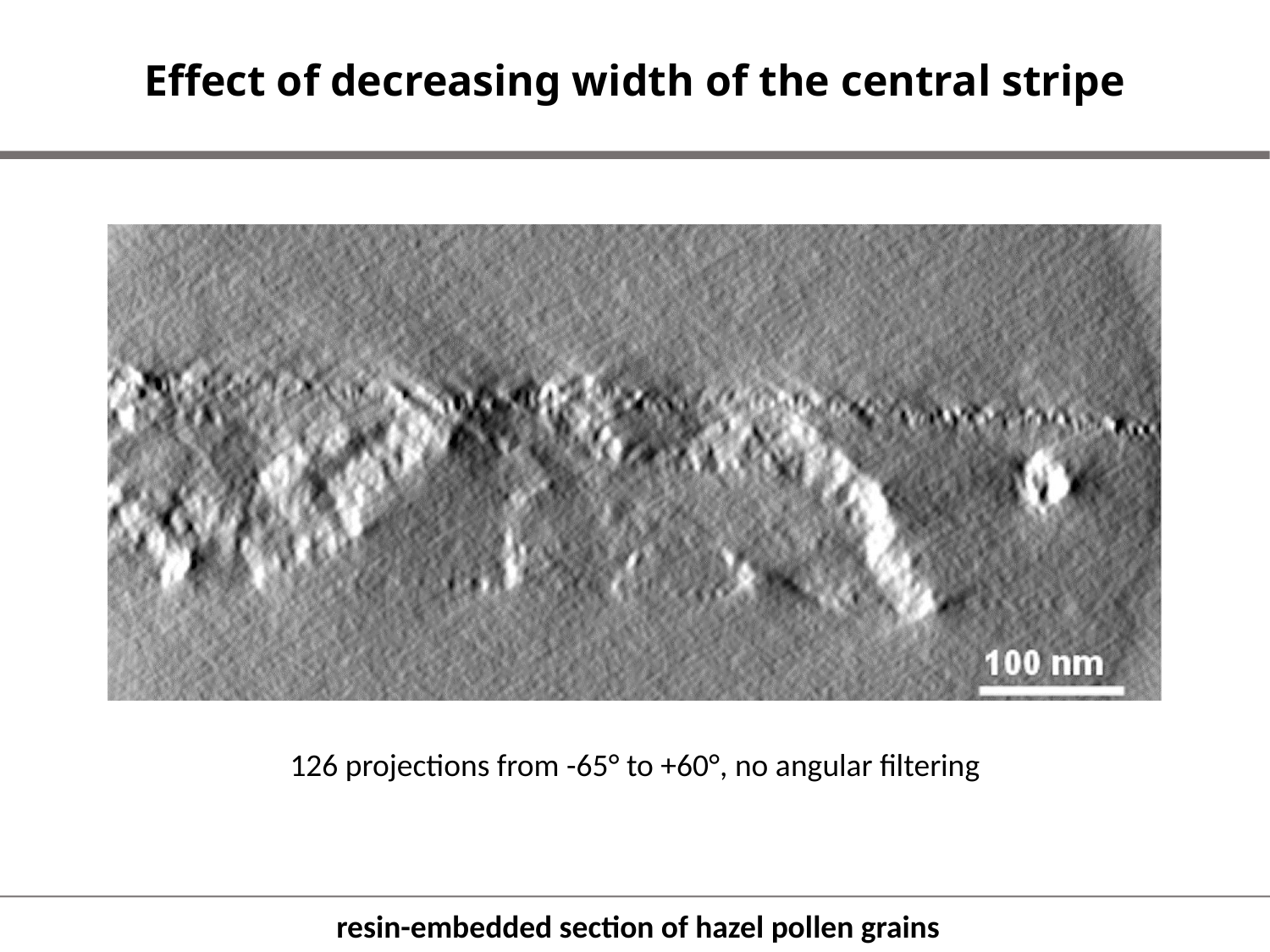

Effect of decreasing width of the central stripe
126 projections from -65° to +60°, no angular filtering
 resin-embedded section of hazel pollen grains

## Slide 6
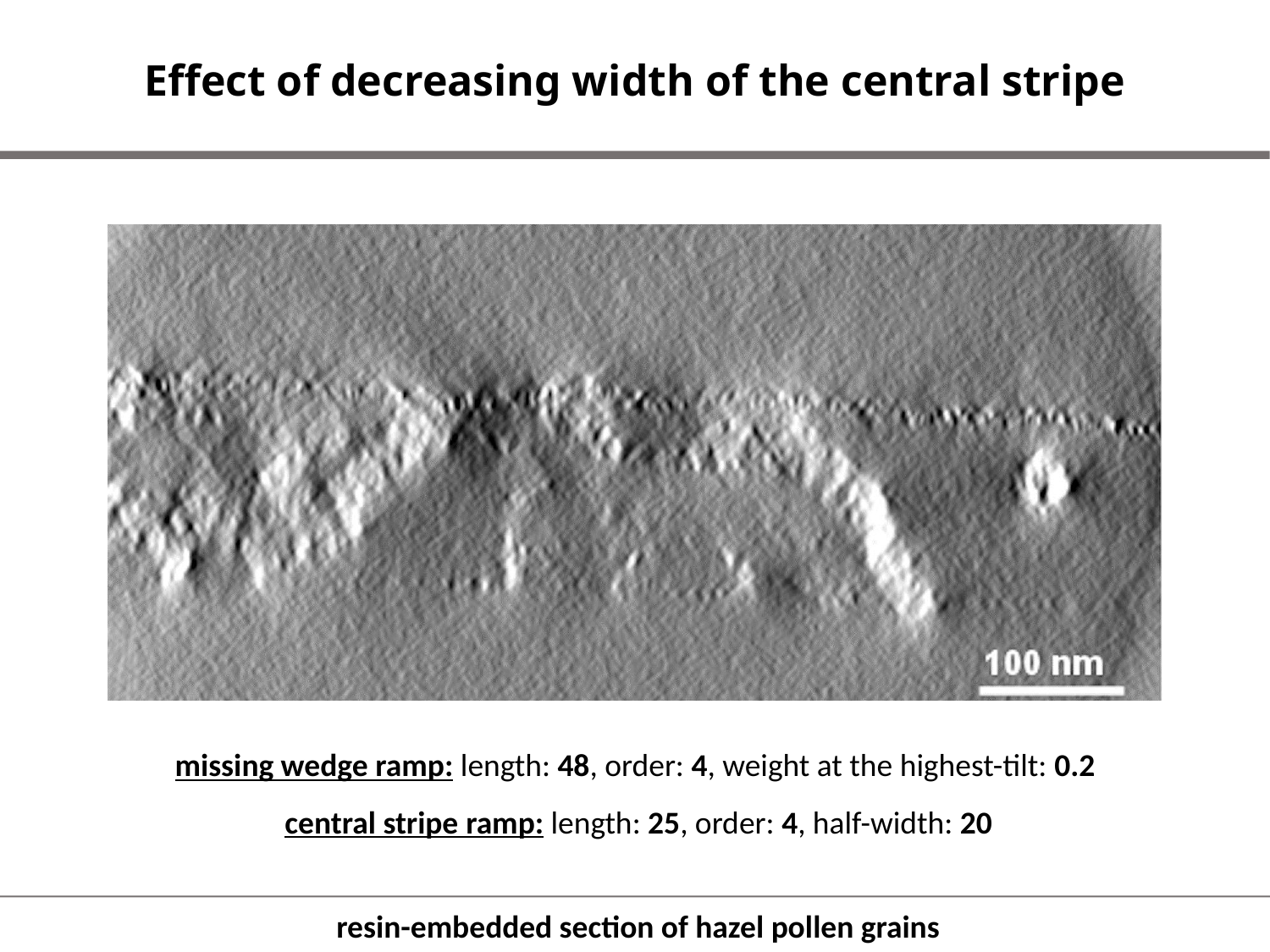

Effect of decreasing width of the central stripe
missing wedge ramp: length: 48, order: 4, weight at the highest-tilt: 0.2
 central stripe ramp: length: 25, order: 4, half-width: 20
 resin-embedded section of hazel pollen grains

## Slide 7
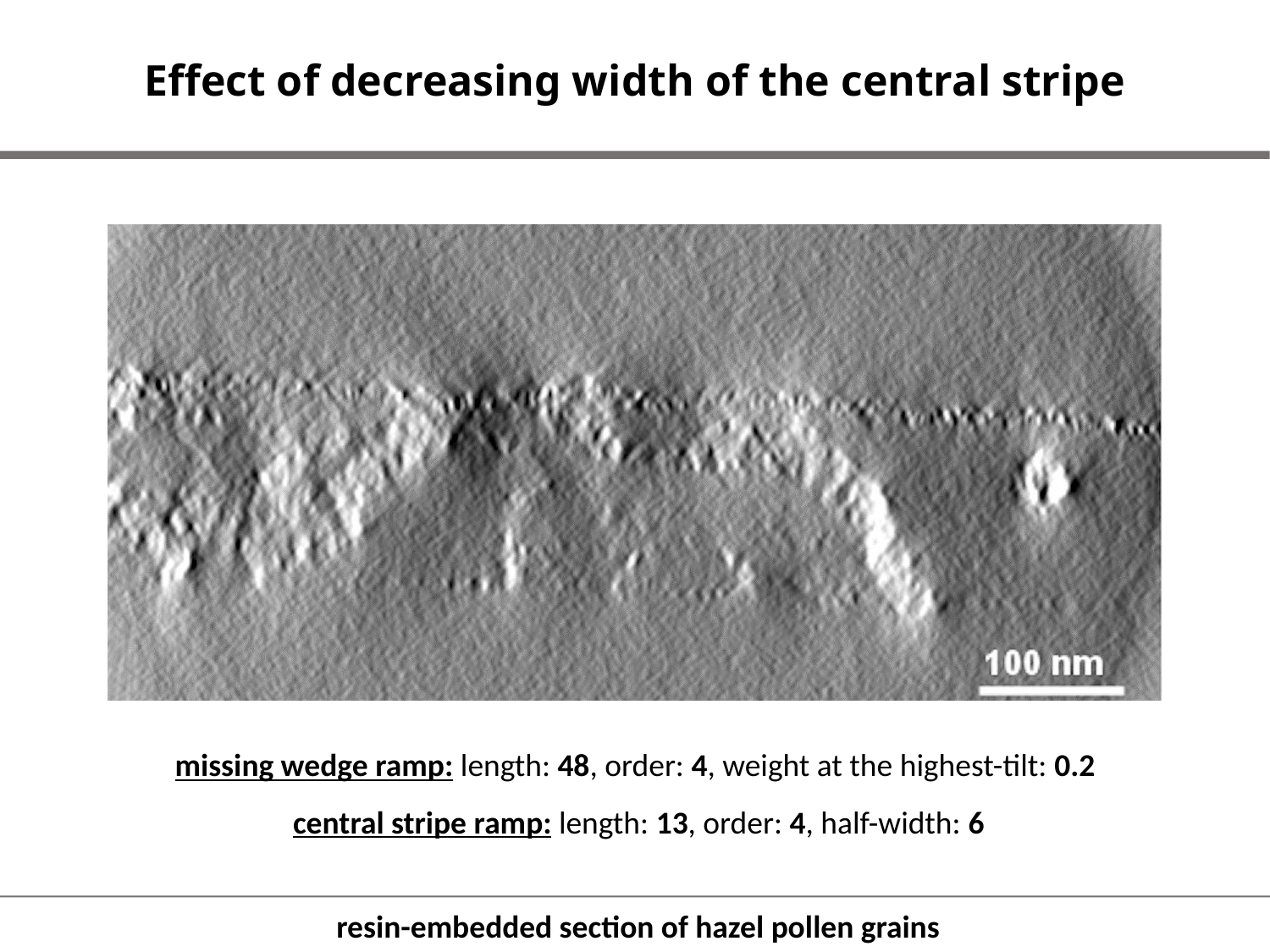

Effect of decreasing width of the central stripe
missing wedge ramp: length: 48, order: 4, weight at the highest-tilt: 0.2
 central stripe ramp: length: 13, order: 4, half-width: 6
 resin-embedded section of hazel pollen grains

## Slide 8
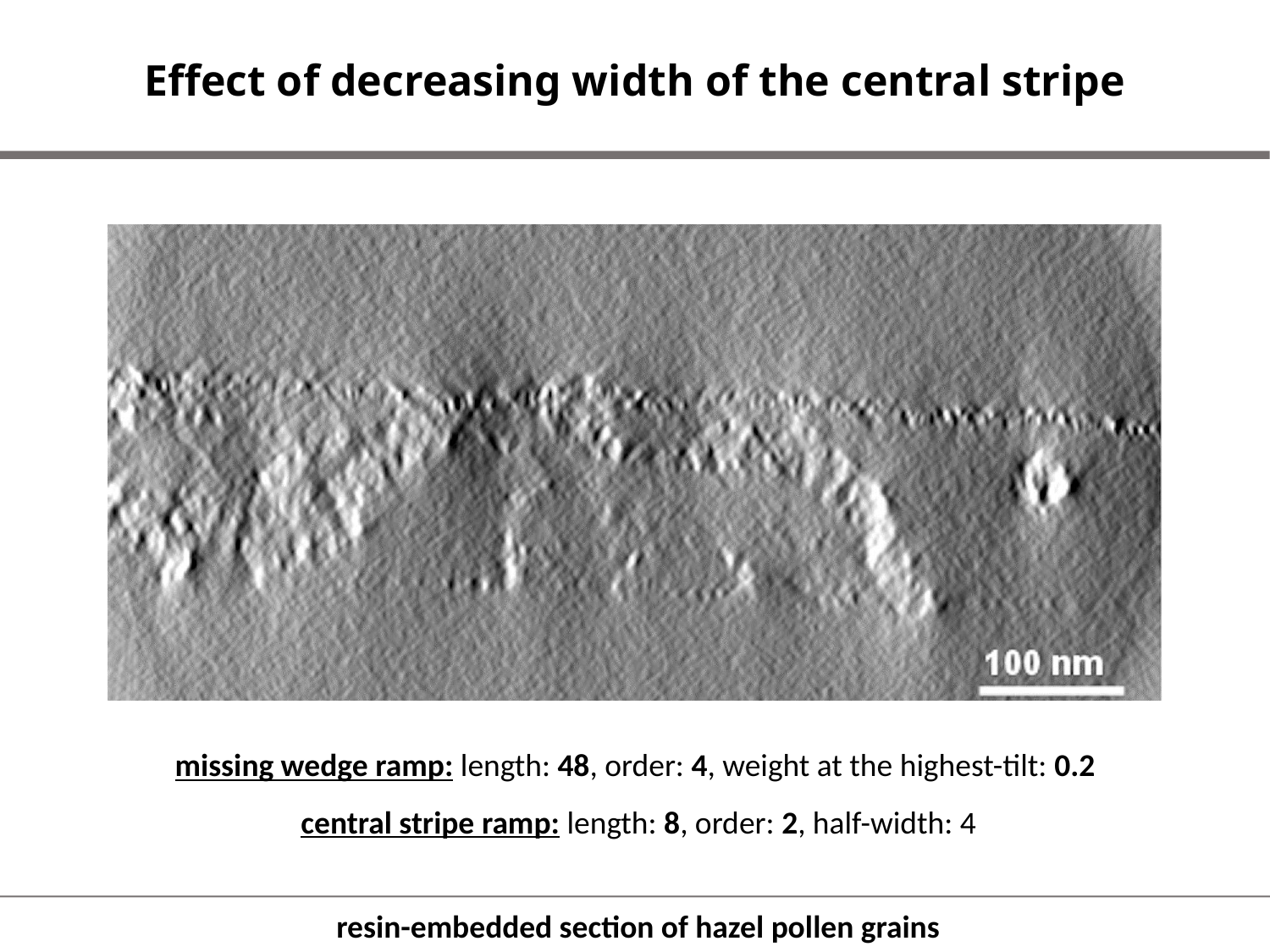

Effect of decreasing width of the central stripe
missing wedge ramp: length: 48, order: 4, weight at the highest-tilt: 0.2
 central stripe ramp: length: 8, order: 2, half-width: 4
 resin-embedded section of hazel pollen grains

## Slide 9
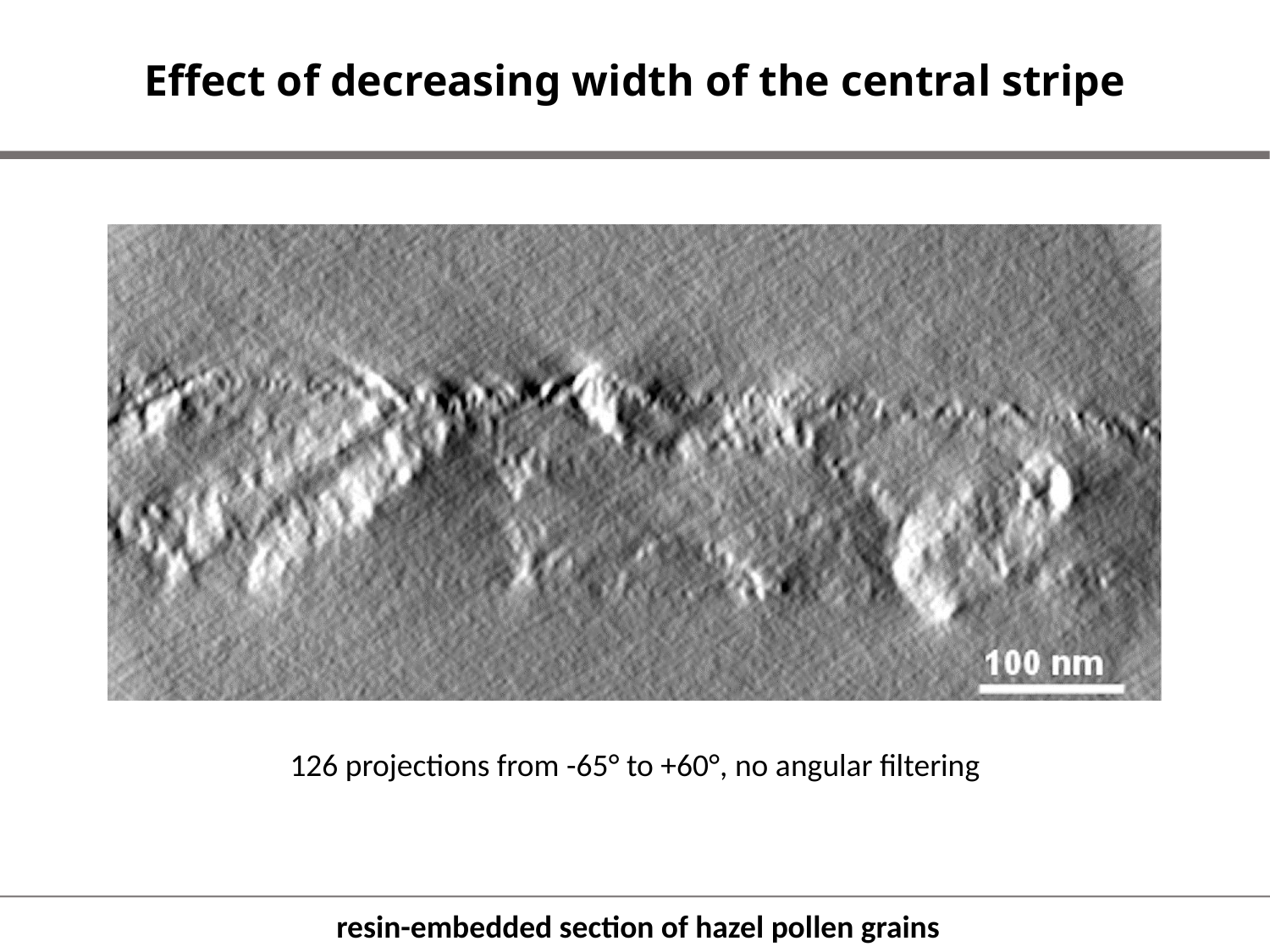

Effect of decreasing width of the central stripe
126 projections from -65° to +60°, no angular filtering
 resin-embedded section of hazel pollen grains

## Slide 10
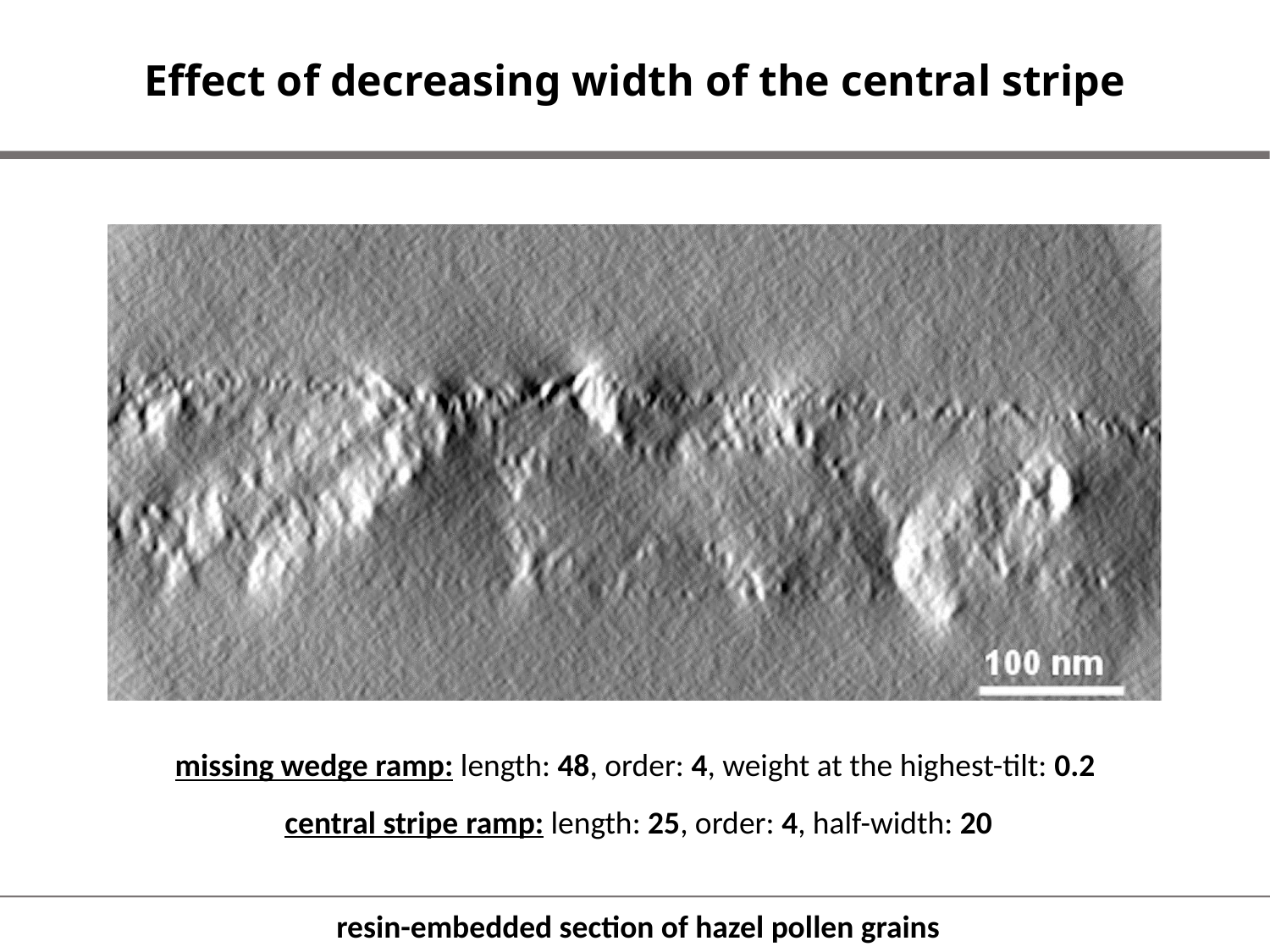

Effect of decreasing width of the central stripe
missing wedge ramp: length: 48, order: 4, weight at the highest-tilt: 0.2
 central stripe ramp: length: 25, order: 4, half-width: 20
 resin-embedded section of hazel pollen grains

## Slide 11
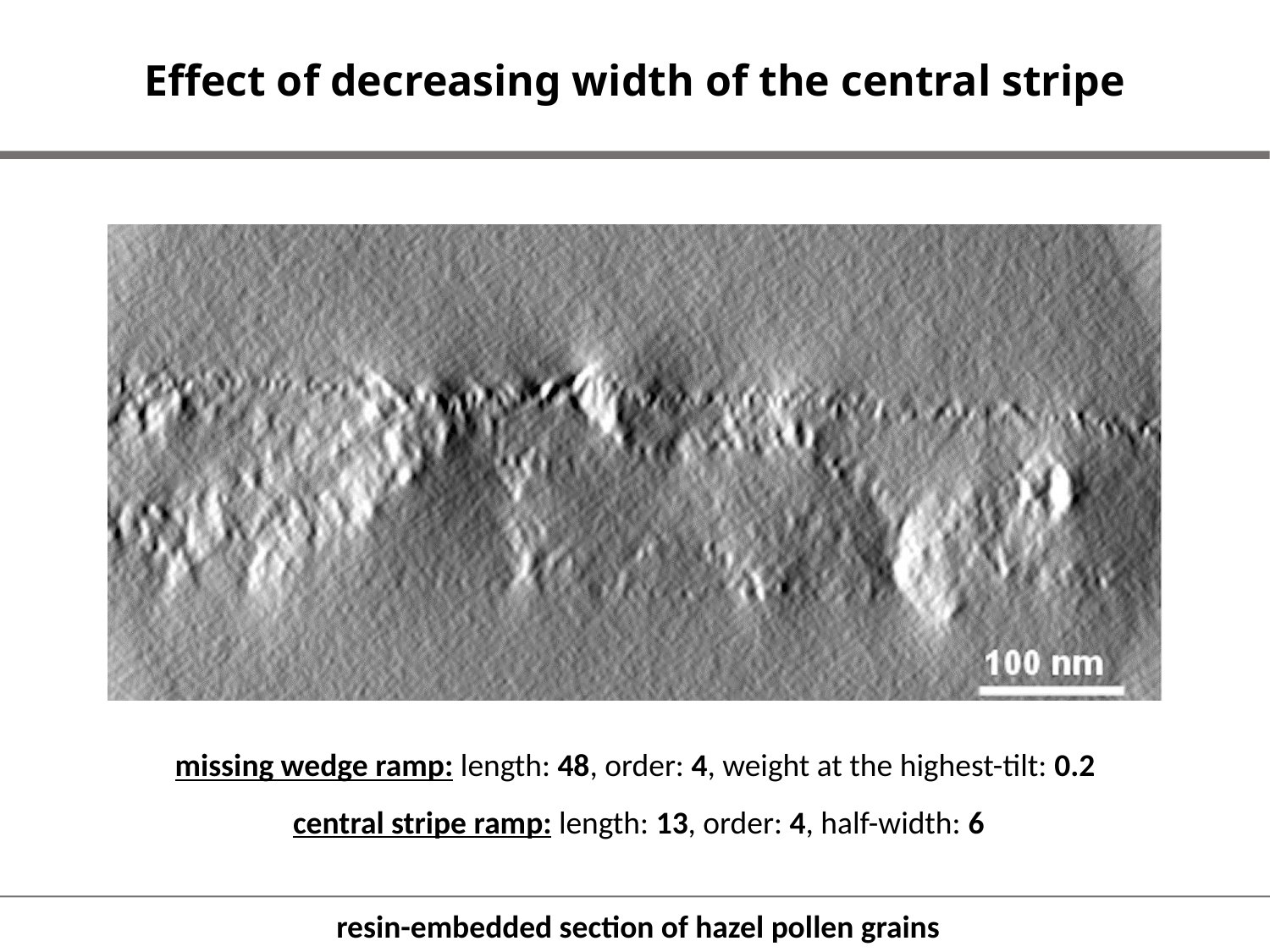

Effect of decreasing width of the central stripe
missing wedge ramp: length: 48, order: 4, weight at the highest-tilt: 0.2
 central stripe ramp: length: 13, order: 4, half-width: 6
 resin-embedded section of hazel pollen grains

## Slide 12
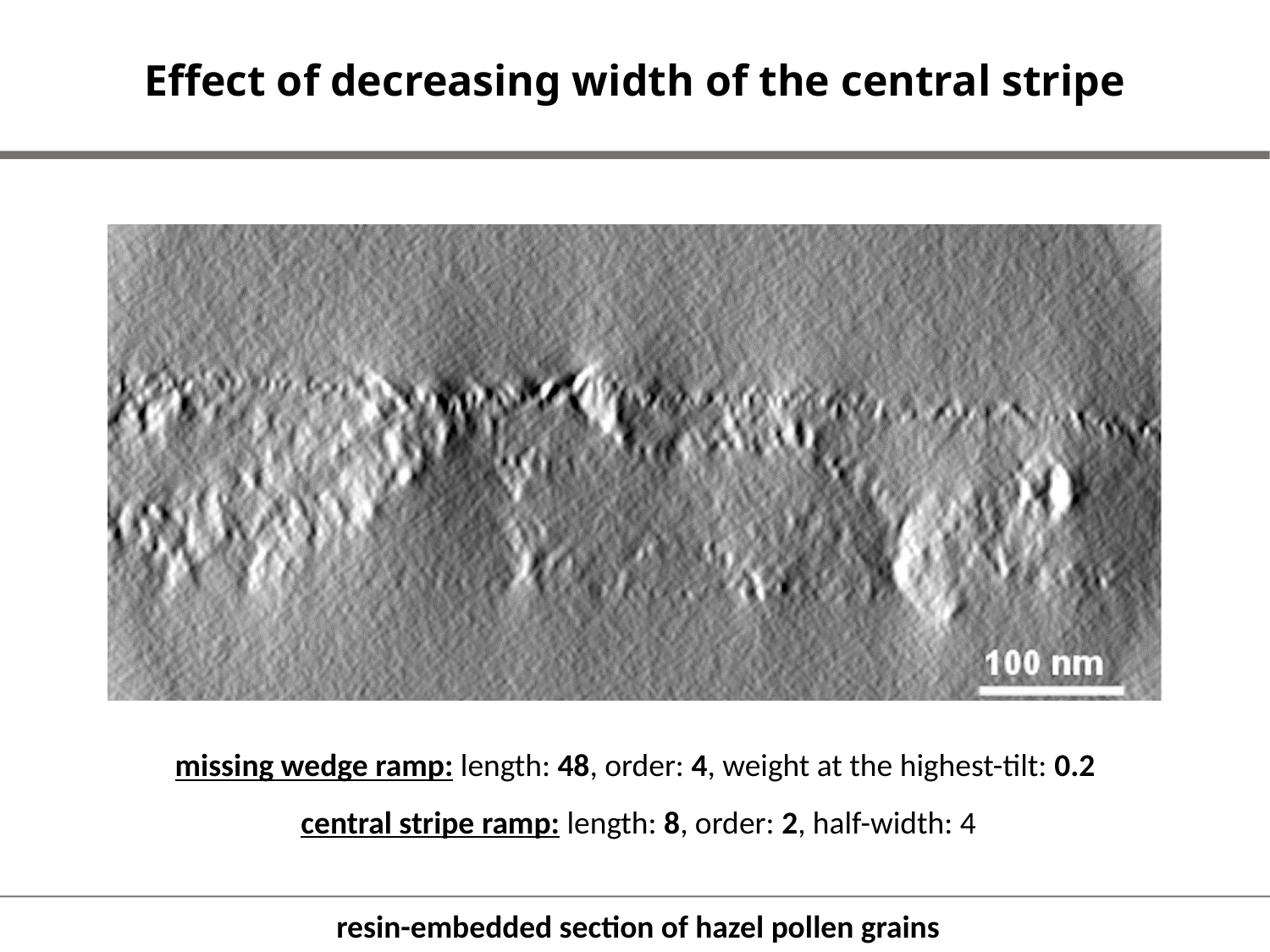

Effect of decreasing width of the central stripe
missing wedge ramp: length: 48, order: 4, weight at the highest-tilt: 0.2
 central stripe ramp: length: 8, order: 2, half-width: 4
 resin-embedded section of hazel pollen grains
