# Supplementary material for: A simple Fourier filter for suppression of the missing wedge ray artefacts in single-axis electron tomographic reconstructions
Source: J Struct Biol. 2014 Apr;186(1):141–52. doi: 10.1016/j.jsb.2014.02.004 (PMC3991334; doi:10.1016/j.jsb.2014.02.004)
Supplement: Supplementary data 5 — This document file contains Supplementary Movie 5. [file mmc5.pptx]

## Slide 1
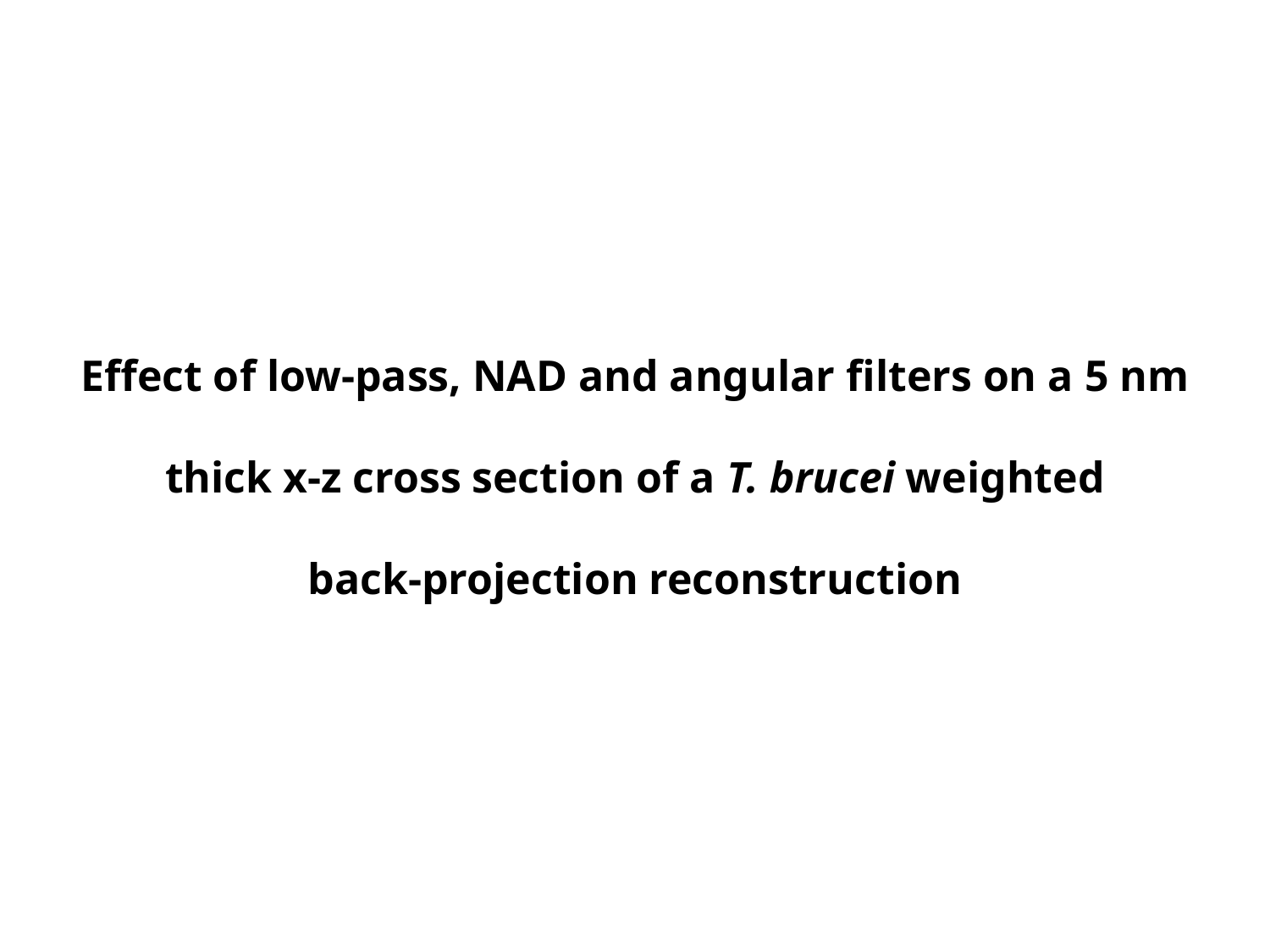

Effect of low-pass, NAD and angular filters on a 5 nm
 thick x-z cross section of a T. brucei weighted
back-projection reconstruction

## Slide 2
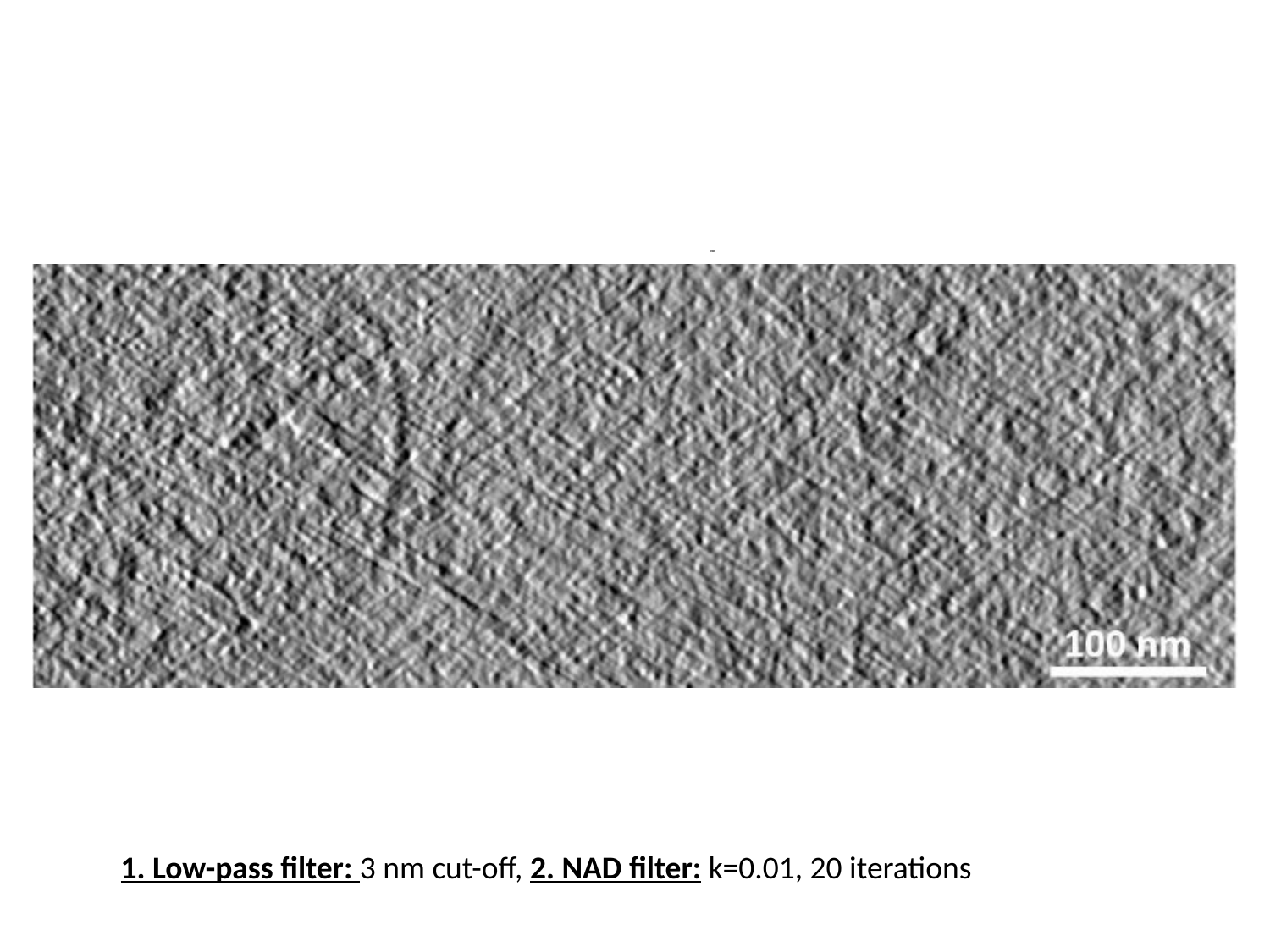

1. Low-pass filter: 3 nm cut-off, 2. NAD filter: k=0.01, 20 iterations

## Slide 3
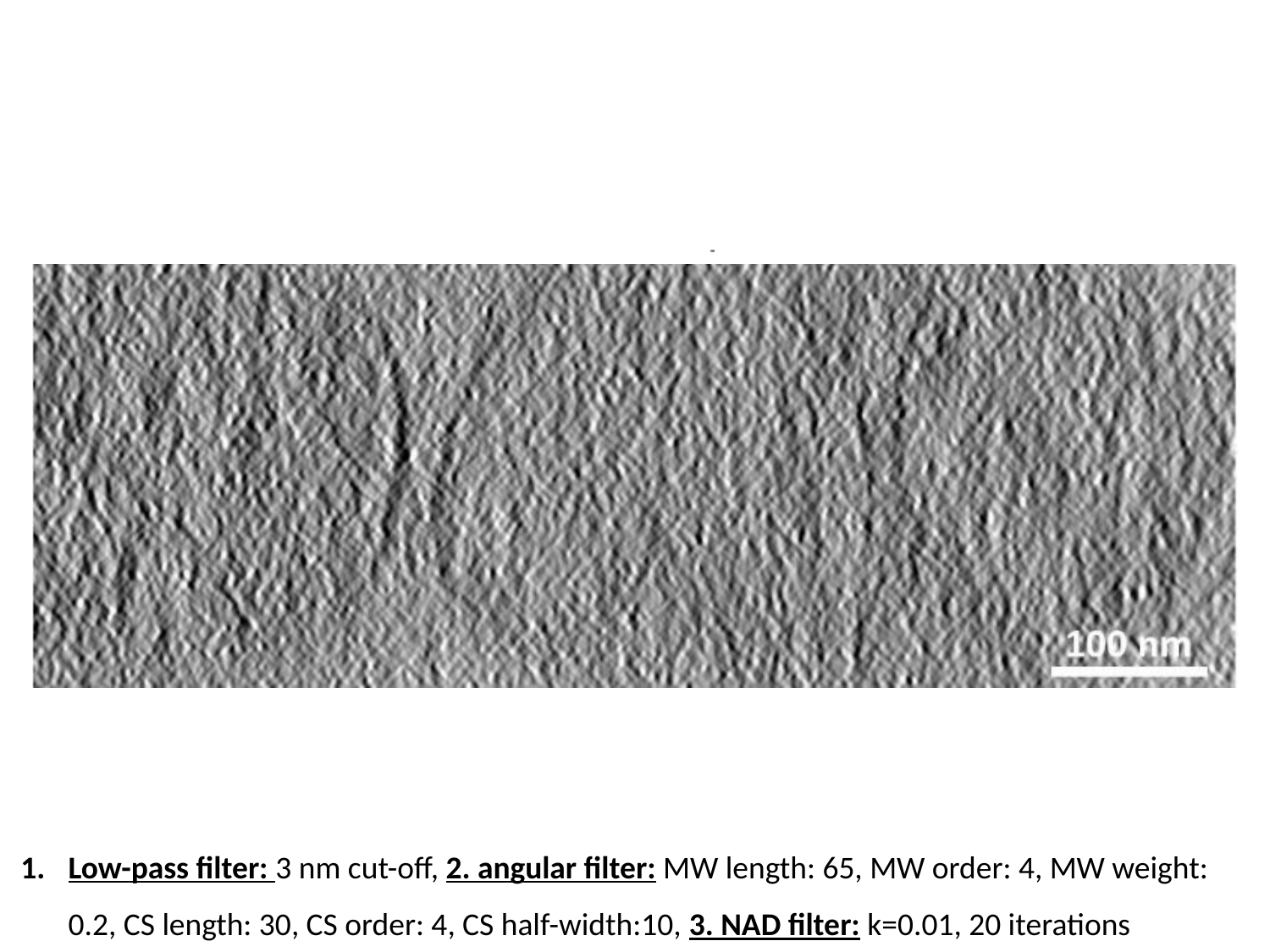

Low-pass filter: 3 nm cut-off, 2. angular filter: MW length: 65, MW order: 4, MW weight: 0.2, CS length: 30, CS order: 4, CS half-width:10, 3. NAD filter: k=0.01, 20 iterations
