# Supplementary material for: A simple Fourier filter for suppression of the missing wedge ray artefacts in single-axis electron tomographic reconstructions
Source: J Struct Biol. 2014 Apr;186(1):141–52. doi: 10.1016/j.jsb.2014.02.004 (PMC3991334; doi:10.1016/j.jsb.2014.02.004)
Supplement: Supplementary data 7 — This document file contains Supplementary Table 2. [file mmc7.pdf]

Supplementary table 2. Performance of angular filtering of volumes containing random knots having gaussian intensity profile cross-sections and cylinders of higher intensity, expressed by mean relevancies of improvement of FOMs. In case of the foreground mean separability FOMs and SNR in reconstructions, simple ratio of angularly filtered versus unfiltered FOMs or SNRs, respectively, was used. Statistically significant improvement is depicted by numbers in bold, inferior performance by standard font, and insignificant changes in performance by underlined italic font. Filters in rows 1,2 and 3 demonstrate the effect of a decreasing weight of the missing wedge ramp at the highest-tilt projection, filters in rows 4, 2 and 5 the effect of narrowing of the central stripe, and filters in rows 6, 2 and 7 the effect of an increasing length of the missing wedge ramp.

		**Whole volume eFOM**						**Signal voxels eFOM**						**Background voxels eFOM**					
		SNR in projections						SNR in projections						SNR in projections					
	**angular filter**	**0.01**	**0.1**	**0.5**	**1**	**5**	**∞**	**0.01**	**0.1**	**0.5**	**1**	**5**	**∞**	**0.01**	**0.1**	**0.5**	**1**	**5**	**∞**
**1**	bfly20-4-0.5-15-4-10	**3.02**	**3.02**	**2.82**	**2.48**	-1.86	-3.89	**2.86**	**2.80**	**1.25**	-1.42	-7.30	-8.28	**3.03**	**3.05**	**3.08**	**3.32**	**5.28**	**7.60**
**2**	bfly20-4-0.2-15-4-10	**8.09**	**8.10**	**7.56**	**6.54**	-5.25	-10.88	**7.76**	**7.61**	**3.84**	-2.77	-16.99	-19.30	**8.13**	**8.17**	**8.16**	**8.56**	**10.16**	**11.16**
**3**	bfly20-4-0.13-15-4-10	**11.42**	**11.44**	**10.69**	**9.23**	-7.25	-15.31	**10.99**	**10.80**	**5.75**	-3.15	-22.22	-25.35	**11.47**	**11.53**	**11.48**	**11.91**	**12.41**	**10.93**
**4**	bfly20-4-0.2-25-4-20	**7.61**	**7.63**	**7.30**	**6.82**	*-0.04*	-3.06	**7.38**	**7.32**	**5.40**	**2.12**	-5.31	-6.21	**7.63**	**7.68**	**7.60**	**7.84**	**6.86**	**5.17**
**5**	bfly20-4-0.2-8-2-4	**8.43**	**8.43**	**7.56**	**5.67**	-13.58	-23.60	**8.03**	**7.75**	**1.14**	-10.59	-34.84	-39.92	**8.47**	**8.52**	**8.58**	**9.19**	**14.29**	**19.21**
**6**	bfly10-4-0.2-15-4-10	**4.60**	**4.61**	**4.27**	**3.63**	-3.88	-7.35	**4.39**	**4.30**	**1.85**	-2.45	-11.67	-13.19	**4.63**	**4.66**	**4.66**	**4.94**	**6.36**	**7.95**
**7**	bfly40-4-0.2-15-4-10	**16.01**	**16.06**	**15.16**	**13.33**	-6.92	-16.79	**15.48**	**15.27**	**9.23**	-1.47	-24.44	-28.37	**16.08**	**16.17**	**16.10**	**16.54**	**16.07**	**13.51**

		**range FOM**						**Signal voxels std. deviation FOM**						**Background voxels std. deviation FOM**					
		SNR in projections						SNR in projections						SNR in projections					
	**angular filter**	**0.01**	**0.1**	**0.5**	**1**	**5**	**∞**	**0.01**	**0.1**	**0.5**	**1**	**5**	**∞**	**0.01**	**0.1**	**0.5**	**1**	**5**	**∞**
**1**	bfly20-4-0.5-15-4-10	**1.49**	**1.57**	**4.14**	-2.26	-3.84	-2.59	**1.47**	**1.69**	**5.26**	-38.89	-8.08	-8.03	**1.53**	**1.54**	**1.55**	**1.68**	**2.72**	**4.00**
**2**	bfly20-4-0.2-15-4-10	**4.48**	**4.12**	**9.51**	-3.84	-7.77	-4.31	**4.03**	**4.62**	**13.57**	-130.62	-18.98	-18.95	**4.15**	**4.17**	**4.17**	**4.38**	**5.28**	**5.98**
**3**	bfly20-4-0.13-15-4-10	**6.62**	**5.93**	**13.58**	-4.01	-8.92	*-3.46*	**5.75**	**6.60**	**18.76**	-185.50	-24.68	-24.73	**5.91**	**5.94**	**5.92**	**6.15**	**6.48**	**5.74**
**4**	bfly20-4-0.2-25-4-20	**4.29**	**3.78**	**7.08**	*2.60*	-0.15	**3.61**	**3.82**	**4.35**	**10.29**	-62.39	-7.61	-7.36	**3.89**	**3.91**	**3.88**	**4.00**	**3.51**	**2.60**
**5**	bfly20-4-0.2-8-2-4	**4.64**	**4.85**	**8.88**	-17.25	-21.45	-19.58	**4.17**	**4.84**	**17.07**	-210.28	-32.41	-32.81	**4.33**	**4.36**	**4.39**	**4.73**	**7.75**	**11.29**
**6**	bfly10-4-0.2-15-4-10	**2.51**	**2.35**	**5.82**	-3.04	-5.44	-3.59	**2.26**	**2.60**	**7.92**	-66.19	-11.77	-11.67	**2.34**	**2.36**	**2.36**	**2.50**	**3.29**	**4.23**
**7**	bfly40-4-0.2-15-4-10	**8.84**	**9.29**	**14.54**	-5.18	-9.72	-2.80	**8.20**	**9.40**	**25.34**	-244.01	-29.20	-29.52	**8.39**	**8.44**	**8.41**	**8.65**	**8.47**	**7.34**

		**Foreground mean separability FOM**						**detectability error FOM**						**SNR**					
		SNR in projections						SNR in projections						SNR in projections					
	**angular filter**	**0.01**	**0.1**	**0.5**	**1**	**5**	**∞**	**0.01**	**0.1**	**0.5**	**1**	**5**	**∞**	**0.01**	**0.1**	**0.5**	**1**	**5**	**∞**
**1**	bfly20-4-0.5-15-4-10	*0.97*	0.99	0.99	**1.00**	**1.01**	**1.01**	0.00	0.00	-0.03	-0.34	-1.02	**0.73**	*0.99*	**1.00**	**1.00**	**1.01**	**1.03**	**1.06**
**2**	bfly20-4-0.2-15-4-10	*0.99*	*1.00*	*1.00*	**1.03**	**1.07**	**1.06**	0.00	0.00	-0.05	-0.59	-2.32	-1.38	*1.04*	**1.05**	**1.04**	**1.05**	**1.07**	**1.09**
**3**	bfly20-4-0.13-15-4-10	*1.01*	**1.02**	**1.02**	**1.05**	**1.11**	**1.10**	0.00	0.00	-0.05	-0.62	-2.84	-2.87	**1.08**	**1.09**	**1.08**	**1.09**	**1.10**	**1.09**
**4**	bfly20-4-0.2-25-4-20	**1.04**	**1.04**	**1.04**	**1.04**	**1.04**	**1.04**	0.00	0.00	**0.03**	**0.26**	**0.58**	**0.59**	**1.09**	**1.08**	**1.08**	**1.08**	**1.07**	**1.05**
**5**	bfly20-4-0.2-8-2-4	0.79	0.85	0.86	0.90	0.98	0.97	0.00	0.00	-0.17	-2.11	-9.64	-6.49	0.85	0.91	0.90	0.92	0.98	1.07
**6**	bfly10-4-0.2-15-4-10	*0.98*	0.99	0.99	**1.01**	1.03	1.03	0.00	0.00	-0.04	-0.44	-1.74	*-0.71*	*1.01*	**1.02**	**1.01**	**1.02**	**1.04**	**1.06**
**7**	bfly40-4-0.2-15-4-10	*1.05*	**1.05**	**1.05**	**1.08**	**1.14**	**1.13**	0.00	0.00	-0.04	-0.44	-2.27	-2.89	**1.15**	**1.15**	**1.14**	**1.16**	**1.15**	**1.13**
